# Evidence for dual targeting control of Arabidopsis 6-phosphogluconate dehydrogenase isoforms by N-terminal phosphorylation

**DOI:** 10.1093/jxb/erae077

**Published:** 2024-02-27

**Authors:** Lennart Nico Doering, Niklas Gerling, Loreen Linnenbrügger, Hannes Lansing, Marie-Christin Baune, Kerstin Fischer, Antje von Schaewen

**Affiliations:** University of Münster, Department of Biology, Institute of Plant Biology and Biotechnology, Molecular Physiology of Plants, Schlossplatz 7, D-48149 Münster, Germany; University of Münster, Department of Biology, Institute of Plant Biology and Biotechnology, Molecular Physiology of Plants, Schlossplatz 7, D-48149 Münster, Germany; University of Münster, Department of Biology, Institute of Plant Biology and Biotechnology, Molecular Physiology of Plants, Schlossplatz 7, D-48149 Münster, Germany; University of Münster, Department of Biology, Institute of Plant Biology and Biotechnology, Molecular Physiology of Plants, Schlossplatz 7, D-48149 Münster, Germany; University of Münster, Department of Biology, Institute of Plant Biology and Biotechnology, Molecular Physiology of Plants, Schlossplatz 7, D-48149 Münster, Germany; University of Münster, Department of Biology, Institute of Plant Biology and Biotechnology, Molecular Physiology of Plants, Schlossplatz 7, D-48149 Münster, Germany; University of Münster, Department of Biology, Institute of Plant Biology and Biotechnology, Molecular Physiology of Plants, Schlossplatz 7, D-48149 Münster, Germany; MPI of Molecular Plant Physiology, Germany

**Keywords:** Arabidopsis PGD2, dual targeting, monomeric import, NADPH provision, OPPP, peroxisomes, protein phosphorylation

## Abstract

The oxidative pentose-phosphate pathway (OPPP) retrieves NADPH from glucose-6-phosphate, which is important in chloroplasts at night and in plastids of heterotrophic tissues. We previously studied how OPPP enzymes may transiently locate to peroxisomes, but how this is achieved for the third enzyme remained unclear. By extending our genetic approach, we demonstrated that Arabidopsis isoform 6-phosphogluconate dehydrogenase 2 (PGD2) is indispensable in peroxisomes during fertilization, and investigated why all PGD–reporter fusions show a mostly cytosolic pattern. A previously published interaction of a plant PGD with thioredoxin *m* was confirmed using Trx_*m2*_ for yeast two-hybrid (Y2H) and bimolecular fluorescent complementation (BiFC) assays, and medial reporter fusions (with both ends accessible) proved to be beneficial for studying peroxisomal targeting of PGD2. Of special importance were phosphomimetic changes at Thr6, resulting in a clear targeting switch to peroxisomes, while a similar change at position Ser7 in PGD1 conferred plastid import. Apparently, efficient subcellular localization can be achieved by activating an unknown kinase, either early after or during translation. N-terminal phosphorylation of PGD2 interfered with dimerization in the cytosol, thus allowing accessibility of the C-terminal peroxisomal targeting signal (PTS1). Notably, we identified amino acid positions that are conserved among plant PGD homologues, with PTS1 motifs first appearing in ferns, suggesting a functional link to fertilization during the evolution of seed plants.

## Teaser

The subcellular localization of the Arabidopsis PGD2 isoform reveals that specific amino acid changes affect targeting to peroxisomes where the enzyme is crucial for fertilization.

## Introduction

The oxidative pentose-phosphate pathway (OPPP) consists of three irreversible reactions that catalyse the conversion of glucose-6-phosphate (G6P) to ribulose-5-phosphate (Ru5P), yielding 2 mol of NADPH at the expense of 1 mol CO_2_. This makes the OPPP an important pathway to generate reduction power from soluble sugars (reviewed in Kruger and von Schaewen, 2003). In plant cells, the OPPP operates as a full cycle only in plastids due to intermediates shared with the Calvin–Benson–Bassham cycle in chloroplasts at night (reviewed in Scheibe, 1990; Buchanan, 1991), which is mandatory in heterotrophic tissues (Neuhaus *et al.*, 1993; Kammerer *et al.*, 1998; Debnam and Emes, 1999). We showed that the irreversible part of the OPPP occurs only transiently in Arabidopsis peroxisomes (Meyer *et al.*, 2011; Hölscher *et al.*, 2014), but is important during fertilization, since heterozygous *PGD2 pgd2* plants produced no homozygous offspring (Hölscher *et al.*, 2016). This was also the case for *GPT1*, encoding one of the two plastidial glucose-6-phosphate transporters (Niewiadomski *et al.*, 2005). We recently demonstrated that GPT1 dually targets plastids and the endoplasmic reticulum (ER), from where the antiporter can be recruited to newly forming peroxisomes under stress to exchange OPPP substrate G6P for Ru5P product (Baune *et al.*, 2020). These unique features may contribute to embryo lethality of *gpt1* but not *gpt2* mutant alleles.

In plant peroxisomes, NADPH is required for the removal of double bonds from hydrocarbons prior to chain shortening via β-oxidation, as in polyunsaturated fatty acids (PUFAs) by 2E,4E-dienoyl-CoA reductase (AtECH2; Goepfert and Poirier, 2007), or during the biosynthesis of phytohormones, such as jasmonic acid (JA) from imported 12-oxophytodienoic acid (OPDA) by oxophytodienoic-acid reductase (AtOPR3; Schaller *et al.*, 2000), and salicylic acid (SA) from imported cinnamic acid by 2,4-dienoyl-CoA reductase (AtSDRb; for a review, see Widhalm and Dudareva, 2015). Also, the formation of nitric oxide (NO) in roots facing abiotic stress, for example induced by sodium chloride (Corpas *et al.*, 2009) or heavy metals (Corpas and Barroso, 2014, 2017), has been linked to NADPH formation in peroxisomes.

Proteins destined for peroxisomes are nuclear encoded and imported fully folded, which is mediated by either a C-terminal peroxisomal targeting signal (PTS)1 or a PTS2 motif within the N-terminus, and recognized by peroxisomal biogenesis factor PEX5 or PEX7, respectively. Both can differ in strength, depending on the amino acid combination (Chowdhary *et al.*, 2012). Peroxisomes show a high turnover, and regularly bud from the ER (Mullen *et al.*, 2001; van der Zand and Tabak, 2013), with the largely oxidizing luminal redox state (confirmed for plant cells; Schwarzländer *et al.*, 2008) needed to promote folding of secretory (glyco)proteins (reviewed in Wang and Wang, 2023). Hence, within nascent peroxisomes, NADPH may be consumed to adjust the glutathione pool (GSH/GSSG) to more reducing conditions via dual cytosolic/peroxisomal glutathione reductase (AtGR1; Kataya and Reumann, 2010). Besides aiding in quenching of reactive oxygen and nitrogen species (ROS and RNS), reduced conditions in the matrix also promote cargo release by a conformational change in PEX5, as has been shown for yeast (Ma *et al.*, 2013) and human cells (Apanasets *et al.*, 2014). Last, but not least, NADPH was shown to be bound by catalase, protecting the enzyme from damage by its substrate H_2_O_2_ and favouring forward reactions (reviewed in Zámocký and Koller, 1999). Yet, NADPH seems to be bound less tightly by plant enzymes, as shown for tobacco catalase (Durner and Klessig, 1996), and NADPH binding is also not supported by sequence data (reviewed in Mhamdi *et al.*, 2012; Palma *et al.*, 2020). In this context, it is interesting that lack of NADH kinase 3 (AtNADK3), that can convert NAD(H) to NADP(H) in peroxisomes, had a negative impact on photorespiration and other metabolic pathways. In addition to glycine and serine, *nadk3* mutant plants accumulated significant levels of G6P, the substrate of the OPPP (Suzuki *et al.*, 2023).

The presence of OPPP reactions in peroxisomes was first proposed upon immunodetecion of glucose-6-phosphate dehydrogenase (G6PDH) in leaf peroxisomes isolated from pea (*Pisum sativum*; Corpas *et al.*, 1998). During the past years, we have examined how OPPP enzymes may be directed to different subcellular locations. First, we studied plastidial G6PD and 6-phosphogluconolactonase (PGL) isoforms of *Arabidopsis thaliana* (Arabidopsis), with our findings suggesting that newly synthesized proteins of the first two enzymes can be diverted from chloroplasts (or heterotrophic plastids) to peroxisomes under conditions linked to oxidative stress or developmental cues. Our proposed model involves interaction of redox-responsive, but catalytically inactive, G6PD4 (At1g09420) with active G6PD1 (At5g35790) in the cytosol, directing the folded G6PD1 precursor to peroxisomes. This interaction seemed to be initiated by a cognate thioredoxin (Trx_*m2*_, At4g03520; Meyer *et al.*, 2011) involved in diurnal regulation of G6PDH activity in the chloroplast stroma. Recently, we showed that alternative splicing of cytosolic *G6PD5* initiates a membrane-bound OPPP metabolon at the cytosolic face of the ER (Linnenbrügger *et al.*, 2022). For the second OPPP step, we found that dual targeting of PGL3 (At5g24400) to plastids and/or peroxisomes is also influenced by Trx_*m2*_, acting as a redox-sensitive co-chaperone with holdase activity in the reduced state but foldase activity in the oxidized state (Hölscher *et al.*, 2014). For the third OPPP step, the situation seemed more straightforward, since 6-phosphogluconate dehydrogenase isoform PGD2 (At3g02360) had been repeatedly identified in peroxisomal proteomes of dark-adapted leaves (Reumann *et al.*, 2007) and etiolated Arabidopsis tissues (Eubel *et al.*, 2008; Quan *et al.*, 2013), but the coding sequence seems not to be influenced by alternative splicing.

The appearance of embryo-defective and pollen-defective genes indicated the significance of OPPP reactions in plastids and/or peroxisomes during reproductive development. Absence of PGL3 activity (emb2024; Meinke *et al.*, 2008) or PGD2 activity (pollen-defective; Niewiadomski *et al.*, 2005), due to central T-DNA insertions, prevented the occurrence of homozygous mutants in different Arabidopsis accessions. Although PGL3 has the ability to target both plastids and peroxisomes (Hölscher *et al.*, 2014), its activity appeared to be primarily required in plastids, based on previous findings (Xiong *et al.*, 2009; Bussell *et al.*, 2013). Importantly, PGD2 does not target plastids (Hölscher *et al.*, 2016; Lutterbey and von Schaewen, 2016), leaving the possibility that its activity may be important in both the cytosol and peroxisomes. However, given the presence of PGD1 and PGD3 in the cytosol and plastids (Hölscher *et al.*, 2016), plus additional redundancy with other NADPH-generating enzymes, such as cytosolic NADP-isocitrate dehydrogenase (NADP-cICDH; Mhamdi *et al.*, 2010), non-phosphorylating glyceraldehyde-3-phosphate dehydrogenase (GAPN; Rius *et al.*, 2006), and several isoforms of NADP-malic enzyme (NADP-ME; Wheeler *et al.*, 2005), it seems unlikely that 6PGDH activity is indispensable in the cytosol. Furthermore, heterozygous *AMC amc* (*ABSTINENCE BY MUTUAL CONSENT* equivalent to *PEX13/APM2*; Boisson-Dernier *et al.*, 2008) mutant lines showed transmission ratios similar to *PGD2 pgd2* (Hölscher *et al.*, 2016), which linked PGD2 to important steps during pollen tube growth and fertilization. Furthermore, this suggested a specific role for PGD2 in peroxisomes that—unlike for the cytosolic situation—cannot be compensated by other NADPH-generating enzymes during fertilization, since only a stomata-opening phenotype was found for peroxisomal NADP-ICDH (At1g54340, Leterrier *et al.*, 2016). Still, reporter–PGD2 fusions (with an unmasked C-terminal PTS1 motif -SKI>) were not imported by peroxisomes in bombarded epidermal onions cells (Reumann *et al.*, 2007) and neither in transfected Arabidopsis mesophyll protoplasts (Hölscher *et al.*, 2016). Only upon N-terminal truncation (Δ1st ATG, lacking 15 amino acids), we found the reporter–PGD2 constructs in peroxisomes, but a corresponding complementation construct did not rescue the fertilization defect—probably due to catalytic inactivity (Hölscher *et al.*, 2016).

Here we show by extended analyses that PGD2 activity is needed inside peroxisomes to overcome the fertilization block, and that all Arabidopsis PGD isoforms can interact with disulfide isomerase Trx_*m2*_. Yet, exchange of unique cysteine residues in the PGD isoforms affected only the speed of peroxisomal import (tested with N-terminally truncated PGD2), but did not improve plastid import of the PGD1 isoform. To investigate peroxisomal targeting of full-length PGD2, we devised a new medial reporter fusion that did not abolish catalytic activity, and investigated the influence of N-terminal truncations and single amino acid changes. These analyses finally hinted at the importance of an N-terminal threonine (Thr6) in PGD2 and a corresponding serine (Ser7) in PGD1/3, right in front of the highly conserved NADP-binding domain (-RIGLAG-). Subsequent phosphomimetic amino acid changes led to exclusive organelle import, but poor protein solubility and undetectable catalytic activity. Taken together, our analyses suggest that early kinase activity is needed to prevent PGD dimer formation in the cytosol and enable organelle import. This can also explain the high conservation of the Thr position in the peroxisomal and of the synonymous Ser position in the plastidial PGD homologues of all flowering plants examined.

## Materials and methods

### Bioinformatics

Information on *Arabidopsis thaliana* was retrieved from The Arabidopsis Information Resource (TAIR) (https://www.arabidopsis.org/) or ARAMEMNON (http://aramemnon.uni-koeln.de/). For sequence alignments, we used Clustal Omega (https://www.ebi.ac.uk/Tools/msa/clustalo/) provided by the EMBL’s European Bioinformatics Institute (Heidelberg, Germany), or the Expasy Translate tool (https://web.expasy.org/translate/) of the Swiss Institute of Bioinformatics. Protein interactions were searched with BioGRID and phosphorylation sites with PhosPhAt 4.0. Protein 3D models are based on AlphaFold predictions and were modified with DNASTAR Protean 3D v17. Phylogenetic trees were built and modified with MEGA (Molecular Evolutionary Genetic Analyses, v11).

### Arabidopsis mutants

Information on Arabidopsis T-DNA insertion lines was retrieved from the SIGnAL database at http://signal.salk.edu (Alonso *et al.*, 2003). Mutant lines were ordered from the Nottingham Arabidopsis Stock Centre (NASC) and analysed by PCR of genomic DNA as suggested for T-DNA insertion lines by TAIR for SALK_036751 (*pgd2-1*; Hölscher *et al.*, 2016).

### Reverse transcription–PCR

Total RNA was isolated from 8-day-old light-grown *A. thaliana* wild-type (Col) seedlings using the NucleoSpin RNA Plant kit (Macherey-Nagel). Reverse transcription to cDNA was done with Superscript II Reverse Transcriptase (Invitrogen) and oligo(dT)_18_ primers (ThermoFisher Scientific) after DNA digestion with RNase-free DNase I (Invitrogen). PCR amplification from cDNA was conducted with 0.1–1 µl of the reverse transcription reaction and appropriate primer combinations (10 µM each) using Taq DNA polymerase (Biozym).

### Plant growth

Seeds of Arabidopsis wild type (var. Columbia) or heterozygous *PGD2 pgd2* mutant plants (including complemented lines) were treated as described in Linnenbrügger *et al.* (2022). Seeds underwent surface sterilization in varying concentrations of ethanol (70% 100, and 70%) and were placed on sterile Arabidopsis germination medium, containing 1% sucrose and 0.5 Murashige and Skoog salts [2.2 g l^–1^ MS salt mix with vitamins, adjusted to pH 5.7−5.8, and 0.8% (w/v) agar; Duchefa Biochemie B.V, Haarlem, The Netherlands]. After stratification, plates were kept under short-day conditions (8 h light at 22 °C and 16 h darkness at 20 °C). For tissue culture, plants were transferred to sterile Magenta boxes (Sigma) with continued cultivation under a short-day regime (9 h light, irradiance 150 µmol m^–2^ s^–1^, at 23 °C and 15 h darkness at 21 °C). For genotyping, plants were transferred to soil and kept under short-day conditions (8 h light, irradiance 150 µmol m^–2^ s^–1^, at 22 °C and 16 h darkness at 20 °C) for ~3–4 weeks.

### Isolation of genomic DNA and characterization of T-DNA insertion lines

Genomic DNA was isolated from 60 mg of leaf tissue as in Hölscher *et al.* (2016). DNA fragments were amplified with Taq-DNA Polymerase (Fermentas), analysed by gel electrophoresis, subcloned, and sequenced to identify the exact position of the T-DNA insertion. T-DNA border fragments were amplified with gene-specific LP or RP primers in combination with the appropriate T-DNA primer (LBa1 or RBa1; listed in Supplementary Table S1).

### Complementation analyses

The entire *PGD2* region was amplified from genomic DNA using Phusion High-Fidelity DNA Polymerase (Finnzymes, ThermoFisher Scientific) and the indicated primer combination (listed in Supplementary Table S1). The genomic *gPGD2* fragment was inserted into *Eco*RV-opened pBluescript SK and cloned in *Escherichia coli* XL1 blue cells (both from Stratagene). The *gPGD2* fragment was released with *Sma*I and *Sal*I, and inserted into the binary vector pGSC1704(HygR) via *Sna*BI and *Sal*I, ligated and cloned in *E. coli* XL1 blue, and re-transformed into *Agrobacterium tumefaciens* strain GV2260 as described in Scharte *et al.* (2009). Floral dip transformation of heterozygous plants was conducted as described by Clough and Bent (1998). Transformed seedlings were selected on MS agar supplemented with 20 µg ml^–1^ hygromycin B (Duchefa Biochemie B.V, Haarlem, The Netherlands), and cultivated further until segregation analyses in the T_2_ generation.

### Yeast two-hybrid analyses

The experiments were essentially performed as described in Meyer *et al.* (2011) using the Trx_*m2*_ constructs, and PGD constructs of Hölscher *et al.* (2016) and Lutterbey and von Schaewen (2016).

### Cloning of fluorescent reporter fusions

Fluorescent reporter fusion constructs were generated as described earlier (Hölscher *et al.*, 2016; Baune *et al.*, 2020; Lansing *et al.*, 2020; Linnenbrügger *et al.*, 2022). An ORF was amplified from either seedling cDNA or existing plasmid constructs, and inserted into the plant expression vectors via compatible restriction sites. All constructs were verified by sequencing. For oligonucleotide primers used in this study, see Supplementary Table S1, with all constructs indicated.

### Site-directed mutagenesis

Amino acid exchanges were introduced at the nucleotide level using a PCR-based site-directed mutagenesis protocol (originally from Stratagene; now QuikChange Site-Directed Mutagenesis Kit, Agilent Technologies) with appropriate primer combinations (Supplementary Table S1) and S7 Fusion High-Fidelity DNA Polymerase (Biozym). All base changes were verified by sequencing.

### Protoplast transfection

Protoplasts were isolated from aseptic Arabidopsis leaves of sugar-adapted plants (if not indicated otherwise) and transfected with plasmid DNA by a polyethylene glycol (PEG) method as described previously (Linnenbrügger *et al.*, 2022). In short, ~500 000 protoplasts were transfected with pre-mixed plasmid DNA in TE buffer (10 mM Tris-EDTA, pH 7−8). Generally, for proteins of interest, 20 µg, and for marker constructs, 5–7 µg of plasmid DNA were used. Samples were incubated at room temperature in the dark and analysed after ~24 h and 48 h.

### Confocal laser scanning microscopy

Localization studies were conducted with a confocal laser scanning microscope (Leica TCS SP5). Settings for excitation/emission wavelengths were 488/490–520 nm for green fluorescent protein (GFP) or yellow fluorescent protein (YFP), and 561/590–620 nm for orange-shifted monomeric red fluorescent protein (OFP), as in Linnenbrügger *et al.* (2022).

### GFP-Trap enrichment

For activity measurements, GFP-fused proteins were first enriched from protoplast extracts using GFP-Trap agarose beads (ChromoTek/Proteintech, Munich, Germany) as described in Linnenbrügger *et al.* (2022). This time, the wash step was repeated twice as recommended by the manufacturer.

### Determination of 6PGDH activity upon GFP-based pull-down from protoplasts

For determination of 6PGDH activities, mesophyll protoplasts were transfected with the same constructs used for the localization studies. Deviating from the procedure described above, five independent transfections were performed per construct, each with 1 million protoplasts and 30 µg of plasmid DNA. After 24 h incubation at room temperature in the dark, individual transfections were combined and the protoplasts harvested (60 *g* at room temperature for 5 min). Protoplast pellets were then lysed and underwent GFP-Trap enrichment (described above).

6PGDH activity was measured according to the method published by Bailey-Serres and Nguyen (1992). The constructs were used for measurement while still bound to the agarose beads. Obtained activities were divided by the signal intensity of bands on immunoblots (quantified with ImageJ) and the activity of the corresponding wild-type version (set to 100%). To prevent cells from settling in the cuvette, a 40% sucrose solution in 50 mM Tris–HCl, pH 7.5 with 0.25 mM NADP and 1 mM MgCl_2_ was used as assay buffer. The reaction was started by addition of 20 mM 6-phosphogluconate. The reduction of NADP to NADPH was measured photometrically at 340 nm at room temperature every 7 s. The slope was evaluated over a linear range of at least 5 min.

### Immunoblot analyses

Immunoblot analyses were performed as described previously in Linnenbrügger *et al.* (2022).

### Determination of 6PGDH activity from *E. coli*

Cloning and analyses of the His-PGD2 variants was analogous to the procedure described in Hölscher *et al.* (2016), using appropriate primers (listed in Supplementary Table S1).

## Results

### Genomic complementation analyses demonstrate that PGD2 activity is required in peroxisomes

**Fig. 1.**
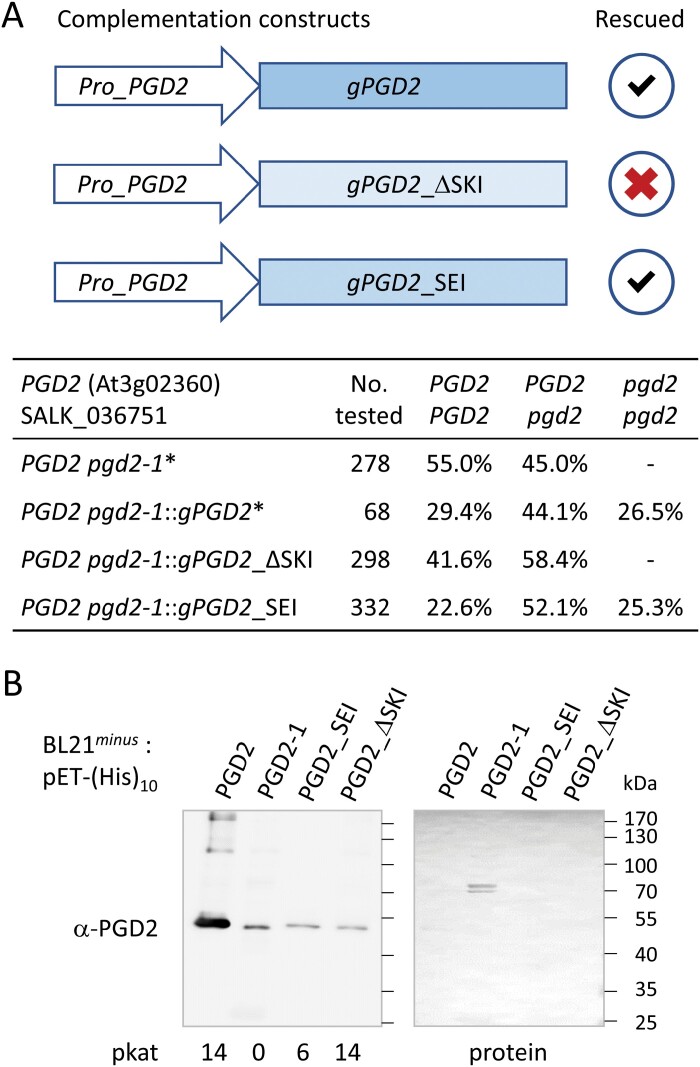
PGD2 activity is needed inside peroxisomes during fertilization. (A) Scheme of genomic complementation constructs and frequency of genotypes for the given transformed *PGD2 pgd2-1* lines (SALK_036751) in the T_2_ generation. No homozygous plants were obtained for the ∆SKI version (dash), even when the corresponding recombinant His-tag protein was expressed in *E. coli* (B), while weak PTS1 (-SEI) sufficed for normal Mendelian distribution. *Pro*, promoter (genomic *PGD2* fragment); Asterisks (*) mark data from Hölscher *et al.* (2016). (B) Activity of the PGD2 cDNA variants cloned in pET16b and purified from G6PDH-deficient *E. coli* strain BL21^*minus*^ (Meyer *et al*., 2011). Aliquots (5 µl) were used for SDS–PAGE separation and blot transfer. The Ponceau S-stained blot (protein) was developed with PGD2 antiserum (α-PGD; Hölscher *et al.*, 2016) using elution fractions of equivalent signal strength (see Supplementary Fig. S1) for comparison on the same blot; pkat, 6PGDH activity (pmol s^–1^); molecular masses in kDa, PageRuler™ Prestained Protein Ladder (Fermentas).

We showed that absence of homozygous *pgd2* mutants is caused by mutual gametophytic sterility (Hölscher *et al.*, 2016). Since unlike the other two PGD isoforms (PGD1/3), PGD2 displays a canonical PTS1 motif (-SKI>) at its C-terminus, missing 6PGDH activity in peroxisomes may lead to the observed fertilization phenotype. To test the importance of PGD2 in peroxisomes, we cloned genomic *PGD2* versions (*gPGD2*) without (ΔSKI) or with a much weaker, non-canonical PTS1 motif (Chowdhary *et al.*, 2012), since an N-terminally truncated reporter-(Δ15)PGD2-SEI> version was not detected in peroxisomes upon protoplast transfection (PGD2_*C-mature-SEI*; Hölscher *et al.*, 2016). Placed under control of their own promoter, heterozygous *PGD2 pgd2-1* plants (SALK_036751) were transformed with the new genomic constructs. Analysis of the T_2_ generation showed that the *gPGD2*_SEI version sufficed to restore Mendelian distribution (Fig. 1A), whereas *gPGD2*_ΔSKI did not produce homozygous offspring among three independent transgenic lines (with ~100 plants tested for each).

When testing 6PGDH activity of corresponding cDNA constructs with a His-tag upon affinity purification from *E. coli* (Supplementary Fig. S1), His-PGD2_ΔSKI showed even higher *in vitro* activity than the PGD2 wild type (Fig. 1B). This led us to conclude that the fertilization phenotype is not caused by lack of PGD2 activity in the cytosol, but specifically inside peroxisomes, which raised questions about the import mechanism and its potential regulation.

### All Arabidopsis PGD isoforms can interact with thioredoxin *m2*

**Fig. 2.**
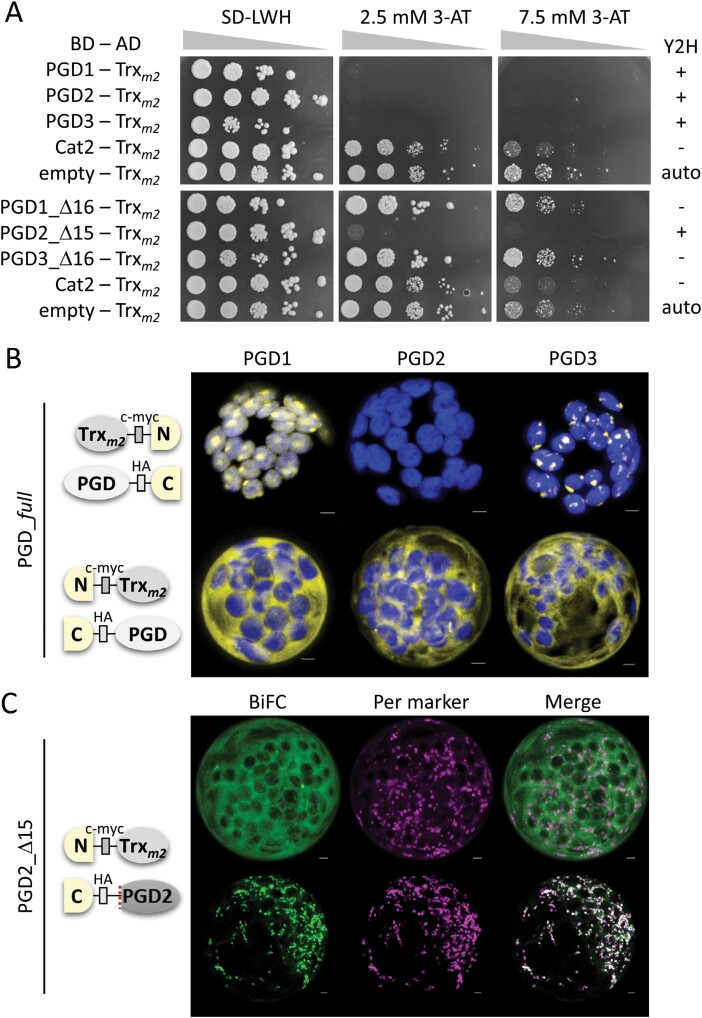
All PGD isoforms interact with thioredoxin *m2* in yeast and plant cells. (A) Yeast two-hybrid assay of the indicated binding and activation domain (BD, AD) combinations in strain SMY3. With the empty vector, Trx_*m2*_ showed autoactivation (auto; Meyer *et al.*, 2011) that was revoked by all PGD isoforms (+, top), but among the N-terminally truncated versions only by PGD2_Δ15 (bottom). Cat2, catalase 2 (main Arabidopsis isoform of green tissues) served as negative control. (B and C) Bimolecular fluorescent complementation (BiFC) analyses in Arabidopsis protoplasts isolated from leaves (48 h post-transfection). (B) Trx_*m2*_ combined with the PGD isoforms; top, with C-terminal split YFP; bottom, with N-terminal split YFP (yellow); chlorophyll autofluorescence is in blue. (C) With N-terminal split YFP, Trx_*m2*_ and PGD2_Δ15 also localized in peroxisomes. All images show maximal projections of ~35 single sections. BiFC is in green and the peroxisomal marker (OFP–PGL3_*C-short*) is in magenta. Co-localization of green and magenta, or very close signals (<200 nm), appear white. Scale bars 3 µm.

We previously showed that interaction with Trx_*m2*_ in the cytosol may direct two plastidial OPPP enzymes to peroxisomes, namely G6PD1 (Meyer *et al.*, 2011) and PGL3 (Hölscher *et al.*, 2014). We therefore investigated if the PGD isoforms may also be capable of interacting with this disulfide isomerase. For yeast two-hybrid (Y2H) assays, the constructs of all PGD isoforms (Lutterbey and von Schaewen, 2016) were tested in combination with those of Trx_*m2*_ (Meyer *et al.*, 2011), using Arabidopsis catalase 2 (Cat2) as control. Drop-out series of different BD–AD combinations revealed that all full-length PGD isoforms interfere with Trx_*m2*_ autoactivation, but not Cat2 (Fig. 2A, top; compare with empty Trx_*m2*_). This is consistent with our previous report on Trx_*m2*_ interaction with PGL3, but not Cat1 (Hölscher *et al.*, 2014). Additional Y2H analyses with N-terminally truncated PGD versions (starting with the second Met at position 16 in PGD2 or 17 in PGD1/3) revealed that only PGD2_Δ15 still interacts with Trx_*m2*_ (Fig. 2A, bottom).

Bimolecular fluorescent complementation (BiFC) of split YFP fusion constructs in Arabidopsis leaf protoplasts supports these results (Fig. 2B; Supplementary Fig. S2). YFP reconstitution was observed for all possible combinations, except when the YFP halves were fused C-terminally to Trx_*m2*_ and PGD2. In the same constellation, PGD1 and PGD3 showed BiFC signals in plastids (Fig. 2B, top). N-terminal fusion of the YFP halves resulted in cytosolic labelling for all three PGD isoforms (Fig. 2B, bottom). Based on the above Y2H results, Trx_*m2*_ interaction was also tested with PGD2_Δ15 (previously termed mature; Hölscher *et al.*, 2016) in co-expression with the peroxisome marker OFP–PGL3_*C-short* (Meyer *et al.*, 2011). Most cells showed cytosolic BiFC signals (Fig. 2C, top), but co-localization with the peroxisome marker was also observed (Fig. 2C, bottom; indicated by mostly white signals in the ‘Merge’ of all channels), although cells with this pattern were infrequently observed. A number of further PGD2–Trx_*m2*_ co-expression experiments, using fusion constructs with an intact fluorescent reporter in different combinations (including a reporter-less Trx_*m2*_ expression cassette in the same vector), did not alter the PGD2 localization pattern noticeably. Thus, for isoforms catalysing the third OPPP step, Trx_*m2*_ binding may occur, but did not allow visible detection of PGD2 inside peroxisomes.

### Unique cysteine residues of the Arabidopsis PGD isoforms are located at the dimer interface

**Fig. 3.**
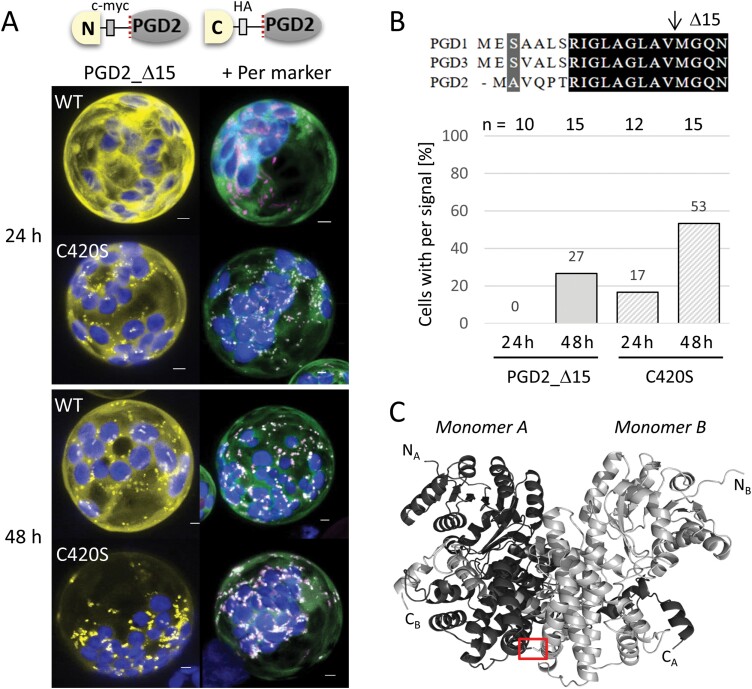
Exchange of the unique cysteine speeds up PGD2 localization in peroxisomes. (A) Bimolecular fluorescence complementation (BiFC) signals of PGD2_Δ15 in Arabidopsis protoplasts isolated from leaves (24–48 h post-transfection). Left panels without and right panels with the peroxisome marker (OFP–PGL3_*C-short*). WT, wild-type PGD2_Δ15; C420S, unique cysteine mutated to serine. All images show maximal projections of ~35 single sections (for single channel images, see Supplementary Fig. S3). BiFC signals are in yellow/green, peroxisomal marker in magenta, and chlorophyll autofluorescence in blue. Co-localization of green and magenta, or very close signals (<200 nm), appear white. Scale bars 3 µm. (B) Frequency of cells with a peroxisomal pattern (% per signal) indicates faster uptake of the C420S version. (C) 3D model of PGD2 (based on X-ray crystallography of the enzyme from sheep; Adams *et al.*, 1991) with unique cysteines on opposite sides of the dimer interface (red frame).

Since interaction with Trx_*m2*_ was possible, a potential role for cysteine C420 (unique to PGD2) was investigated. We chose the split YFP–PGD2_Δ15 versions for further analyses, because they partly localized in peroxisomes. Upon co-transfection with or without the peroxisome marker (to exclude competition for PEX5 binding), the localization pattern was monitored 24 h and 48 h post-transfection (Fig. 3A; Supplementary Fig. S3A). Quantification of cells with a peroxisomal signal suggested that C420S may be more efficiently imported than the wild-type version (Fig. 3B; Supplementary Fig. S3B). Of note, in a three-dimensional (3D) model of PGD2, C420 is located on opposite sides of the two monomers (Fig. 3C, red frame), potentially stabilizing the dimer by a disulfide bond.

To check for similar effects in the plastidial PGD isoforms, the two cysteines unique to PGD1 and PGD3 were exchanged in PGD1–GFP. However, neither C292S nor C435S influenced subcellular localization in transfected Arabidopsis protoplasts, still labelling mostly the cytosol (Supplementary Fig. S4A). Potential effects on catalytic activity were tested with His-Δ6-PGD1 purified from *E. coli*, since longer versions exclusively accumulated in inclusion bodies. Surprisingly, C292S and C435S were about twice as active as the non-mutagenized version (Supplementary Fig. S4B). However, with respect to potential stabilization, only C435 resides on opposite sides of the PGD1 dimer (Supplementary Fig. S4C).

### A new medial GFP fusion of PGD2 is active but does not localize in peroxisomes

**Fig. 4.**
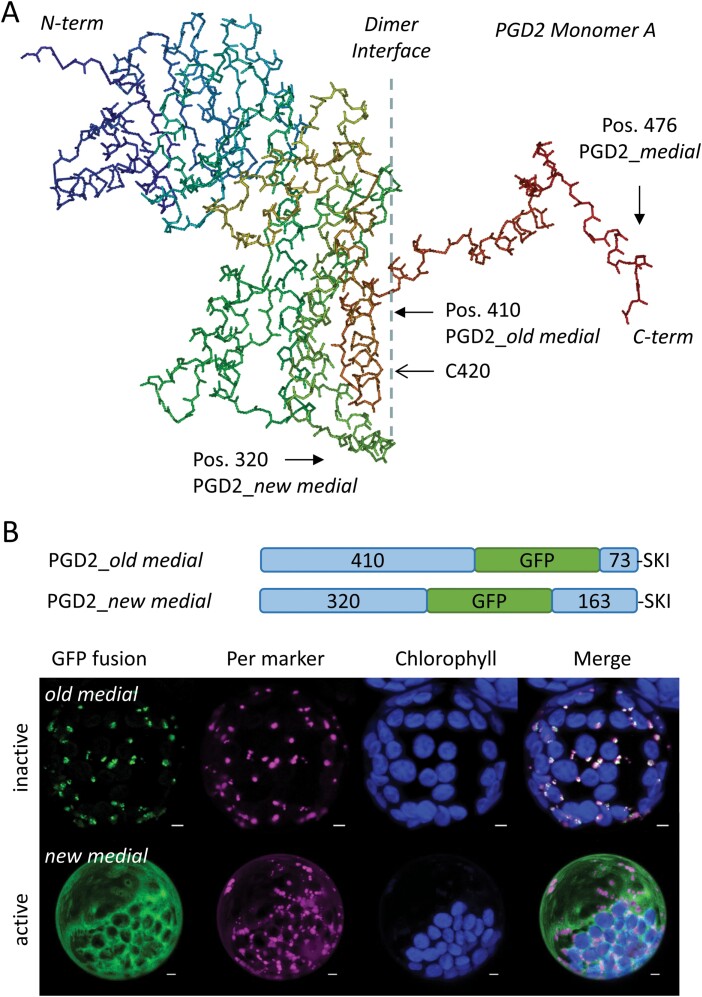
A new medial GFP fusion of PGD2 is active but does not localize in peroxisomes. (A) PGD2 monomer (PDB file obtained from Alphafold, and modifed by Protean 3D) with a large dimer interface (dashed line), the C-terminally extended part with GFP insertion positions (position 476 of Eubel *et al*., 2008), and C420 indicated by arrows. (B) PGD2 constructs with the medial GFP reporter (numbers refer to amino acids of the N- and C-terminal parts) and their analysis in Arabidopsis protoplasts isolated from leaves (48 h post-transfection). Note that *old medial* and *new medial* display opposite localization patterns with an influence on catalytic activity, indicated on the left. All images show maximal projections of ~35 single sections. GFP fusions are in green, peroxisomal marker (OFP–PGL3_*C-short*) in magenta, and chlorophyll autofluorescence in blue. Merge, co-localization of green and magenta, or very close signals (<200 nm), appear white. Scale bars 3 µm.

To investigate peroxisomal targeting of PGD2, we utilized 3D model predictions (AlphaFold; 1 November 2022) to estimate the potential impact of medial GFP insertions. We selected two positions: after amino acid 410 (PGD2_*old medial*) and 320 (PGD2_*new medial*), the latter residing outside the dimer interface (Fig. 4A). These two constructs exhibited completely different localization patterns. While PGD2_*old medial* co-localized with the peroxisome marker, PGD2_*new medial* remained entirely cytosolic. To evaluate a possible influence of the internal reporter on catalytic activity, we performed GFP-based pull-down from transfected Arabidopsis protoplasts. These analyses revealed that cytosolic PGD2_*new medial* is catalytically active, but peroxisome-localized PGD2_*old medial* is not (Fig. 4B). Regarding the more C-terminal insertion position in PGD2_*old medial*, we hypothesized that inactivity may result from interference with dimer formation. This explanation would also apply to the medial construct of Eubel *et al.* (2008) that also localized in peroxisomes, but was not tested for activity.

### Stepwise truncation of the PGD2 N-terminus reveals a crucial amino acid position

**Fig. 5.**
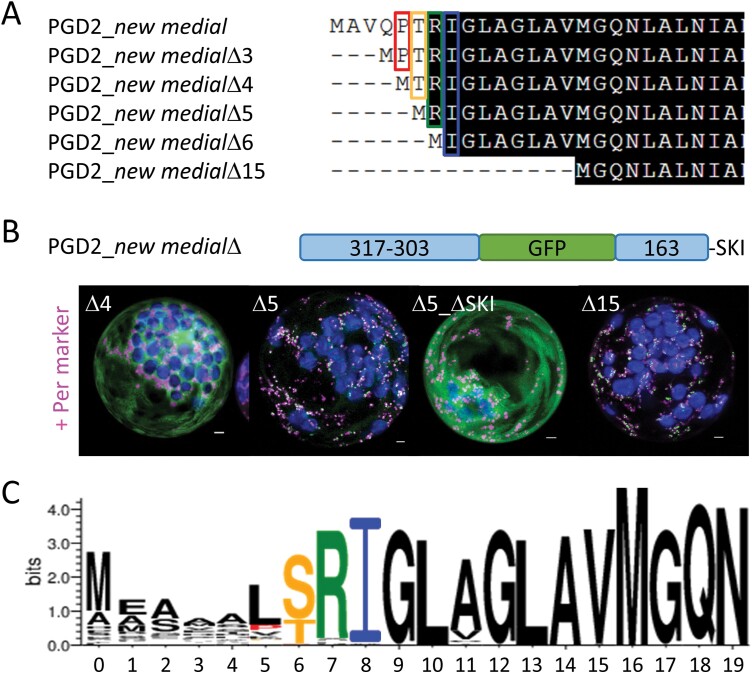
Analysis of N- and C-terminal amino acid deletions in PGD2_*new medial*. (A) Alignment of the PGD2_*new medial* versions with gradual deletion of N-terminal amino acid residues. (B) Localization of N-terminally truncated PGD2_*new medial* versions in Arabidopsis protoplasts isolated from leaves (48 h post-transfection) showed that the border for a clear peroxisomal pattern lies between Δ4 and Δ5 amino acids, hinting at the importance of residue T6 in PGD2. All images show maximal projections of ~35 single sections (merge, for single channel images, see Supplementary Fig. S4). PGD2 fusions are in green, peroxisomal marker (OFP–PGL3_*C-short*) in magenta, and chlorophyll autofluorescence in blue. Co-localization of green and magenta, or very close signals (<200 nm), appear white. Scale bars 3 µm. (C) Logo plot for PGD sequences from different vascular plant clades (61 sequences in total), highlighting amino acid conservation within the N-terminus by letter size. Colors correspond to frames in (A).

As shown before, the N-terminally truncated PGD2_Δ15 version accumulated in peroxisomes, but was inactive due to the absence of highly conserved and probably critical amino acids. This indicated an important role for the PGD2 N-terminus for both activity and targeting. To identify potentially crucial amino acids within the N-terminus, we cloned progressively shorter variants in the PGD2_*new medial* construct (Fig. 5A), lying between full-length PGD2 (active, but cytosolic) and PGD2_Δ15 (inactive, but partly peroxisomal), and co-expressed them with the peroxisomal marker (Fig. 5B). A clear turning point was found, since deletions comprising Thr6 (Δ4, starting <MTRIG- and longer versions) remained uniformly distributed in the cytosol, whereas constructs lacking this residue (Δ5, <MRIG- and shorter versions) co-localized with the peroxisome marker—notably without a cytosolic fraction. For confirmation, we deleted the PTS1 motif (Δ5_ΔSKI), which abolished peroxisomal localization. The majority of PGD homologues from vascular plants display a Thr or Ser at this position (Fig. 5C), which may be subject to phosphorylation.

### Phosphomimetic changes lead to exclusive organelle targeting of the PGD isoforms

**Fig. 6.**
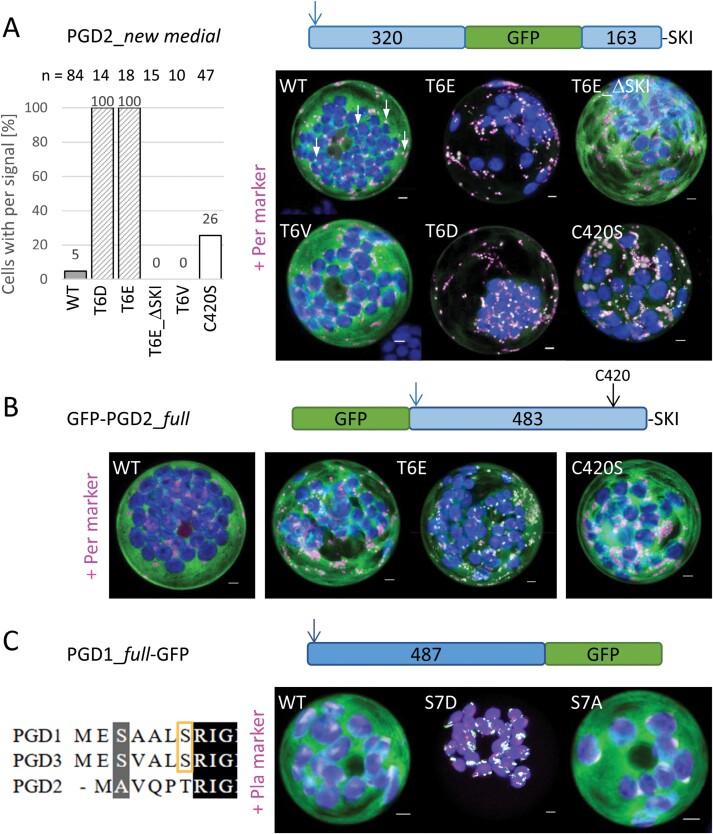
N-terminal phosphomimetic changes promote PGD targeting to organelles. (A) Exchange of T6 to phosphomimetic E or D (but not V or M) in PGD2_*new medial* (arrow) led to a switch in localization (right) from largely cytosolic to a 100% peroxisomal pattern (left). Note that also for the wild tpye (WT) and the C420S version, cells with a partial peroxisomal pattern were scored (white arrows). (B) In GFP–PGD2_*full*, the T6E version showed a partial peroxisomal pattern, suggesting that binding of a cytosolic factor is affected by the N-terminally fused reporter. C420S (black arrow) had no effect. (C) In PGD1_*full*–GFP, exchange of S7 by phosphomimetic D (but not A) led to an exclusive plastidial pattern. All images show maximal projections of ~35 single sections (merge; for single channel images, see Supplementary Fig. S6). GFP fusions are in green, peroxisomal marker (OFP–PGL3_*C-short*) or plastidial marker (GPT2_*5MD*–OFP; Baune *et al.*, 2020) in magenta, and chlorophyll autofluorescence in blue. Co-localization of green and magenta or very close signals (<200 nm) appear white. Scale bars 3 µm.

Based on the previous experiment, we created phosphomimetic changes T6E and T6D via site-directed mutagenesis, including dephosphorylation mimics T6V and T6M as controls. Co-expression with the peroxisomal marker revealed that both T6E and T6D result in peroxisomal localization (with little background), while T6V (Fig. 6) and T6M (Supplementary Fig. S6) remained cytosolic. Notably, the wild-type version also showed some co-localization with the marker in very few cells (WT, indicated by white arrows) that we and others (Reumann *et al.*, 2007) had not observed with N-terminally tagged PGD2 fusions. Quantification of cells with peroxisomal signal revealed co-localization with the marker in <5% of the cells imaged (Fig. 6A, left). Yet, even in these cells, the majority of the signal was cytosolic, while differences between phosphorylation (T6E/T6D, 100%) and dephosphorylation mimics (T6V, 0%) were absolutely clear. For confirmation, we deleted the PTS1 motif (T6E_ΔSKI), which abolished peroxisomal localization. Interestingly, C420S increased the number of cells with a peroxisomal pattern ~5-fold compared with the wild type, but at a much lower frequency than the phosphomimetic changes. Next, we tested T6E and C420S in N-terminally tagged GFP–PGD2_*full*, showing either partial localization in peroxisomes still with cytosolic background, or mostly cytosolic signal for T6E, while C420S alone did not enable peroxisomal import (Fig. 6B), which underlines the importance of a free PGD2 N-terminus.

The corresponding amino acid position in the plastidial isoforms was tested with PGD1. We created S7D and S7A (as non-phosphorylated control) in the C-terminally tagged PGD1_*full*–GFP construct and co-expressed them with a plastidial marker (Fig. 6C). While the wild-type version remained largely cytosolic (with only faint co-localization in the stroma), the phosphomimetic S7D change led to exclusive plastidial localization, with next to no cytosolic background. By contrast, S7A behaved like the wild-type version, with a large cytosolic fraction and only minor signals in the plastid stroma.

### The T6E exchange reduces protein solubility and catalytic activity of PGD2

**Fig. 7.**
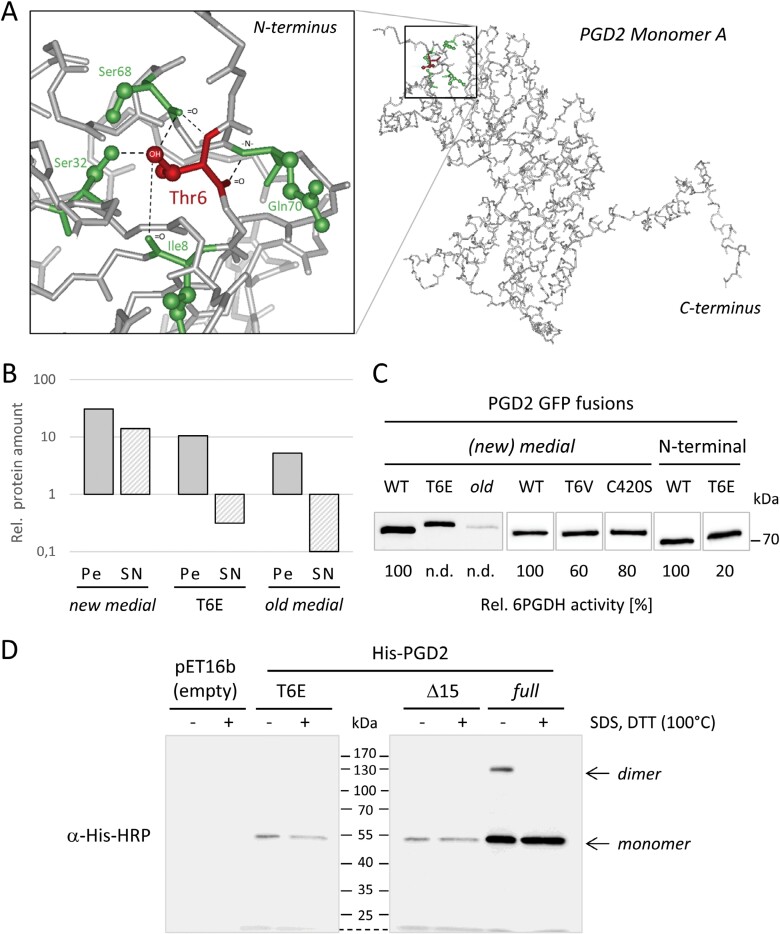
The phosphomimetic change T6E lowers PGD2 solubility and catalytic activity. (A) AlphaFold prediction of intramolecular bonds (within a radius of 5 Å) in the N-terminal part of PGD2. The magnification shows that Thr6 (red) is bound by surrounding Ile8, Ser32, Ser68, and Gln70 (green). (B) Relative protein amounts of PGD2 medial variants compared with endogenous PGD signals in cleared extracts of Arabidopsis protoplasts. Note that compared with PGD2_*new medial*, T6E and *old medial* versions are depleted from the supernatant (SN) but less from the pellet (Pe) fractions, indicative of lower solubility. (C) Relative activity of the indicated PGD2–GFP fusions compared with the corresponding wild type (WT, 100%) upon pull-down from supernatant fractions; n.d., not detected. The immunoblot was developed with anti-PGD2 antiserum (Hölscher *et al.*, 2016). Note that with N-terminal GFP, PGD2-T6E showed catalytic activity. Compilation of different blots (marked by outlines). The lighter lanes stem from the same blot from which empty lanes and the marker were removed. (D) SDS–PAGE of His-PGD2 variants from cleared *E. coli* BL21^*minus*^ extracts without (–) or with (+) SDS and DTT in the sample buffer (plus boiling at 100 °C). The immunoblot was developed with anti-His–HRP conjugate to visualize only recombinant PGD proteins. kDa, PageRuler™ Prestained Protein Ladder (Fermentas).

We inspected the position and intramolecular bonds of Thr6 in the 3D model predicted by AlphaFold (Fig. 7A) to evaluate its importance for catalytic activity and peroxisomal localization. Thr6 engages in bonding with several amino acids (Ile8, Ser32, Ser68, and Gln70), supporting a structural relevance. Most of the interactions seem to be mediated through hydrogen bonds facilitated by the hydroxyl group of threonine, which is absent in the mutagenized T6V and T6M variants. Therefore, selected GFP fusions were subjected to pull-down from transfected Arabidopsis protoplasts and tested for 6PGDH activity (Fig. 7B, C). Compared with PGD2_*new medial*, the T6E and PGD2_*old medial* (used as control) showed little recovery, especially from supernatant fractions, indicative of low solubility (Fig. 7B). This was paralleled by non-detectable activity for T6E and *old medial*, while T6V and C420S showed ~60% and 80% activity, respectively, compared with the corresponding wild-type version. Of note, T6E was still 20% active in N-terminally tagged GFP–PGD2 (Fig. 7C).

To elucidate the reason for partial inactivity, we compared His-tagged T6E and the Δ15 variant to PGD2 wild type upon expression in *E. coli*. SDS–PAGE separation was performed under semi-native conditions, followed by immunoblotting with His-tag-specific antibodies (Fig. 7D). While the full-length protein was active and found in two fractions (migrating as a monomer and dimer, unless boiled in the presence of SDS and DTT_red_), T6E could only be visualized as a monomer, similar to the inactive Δ15 variant (His-PGD2*mat*; Hölscher *et al.*, 2016). This matches the low protein solubility and lack of catalytic activity in Arabidopsis cells (Fig. 7B,C).

### Phylogenetic analyses reveal conservation of the -TRI- and -PTS1> motifs in angiosperms

**Fig. 8.**
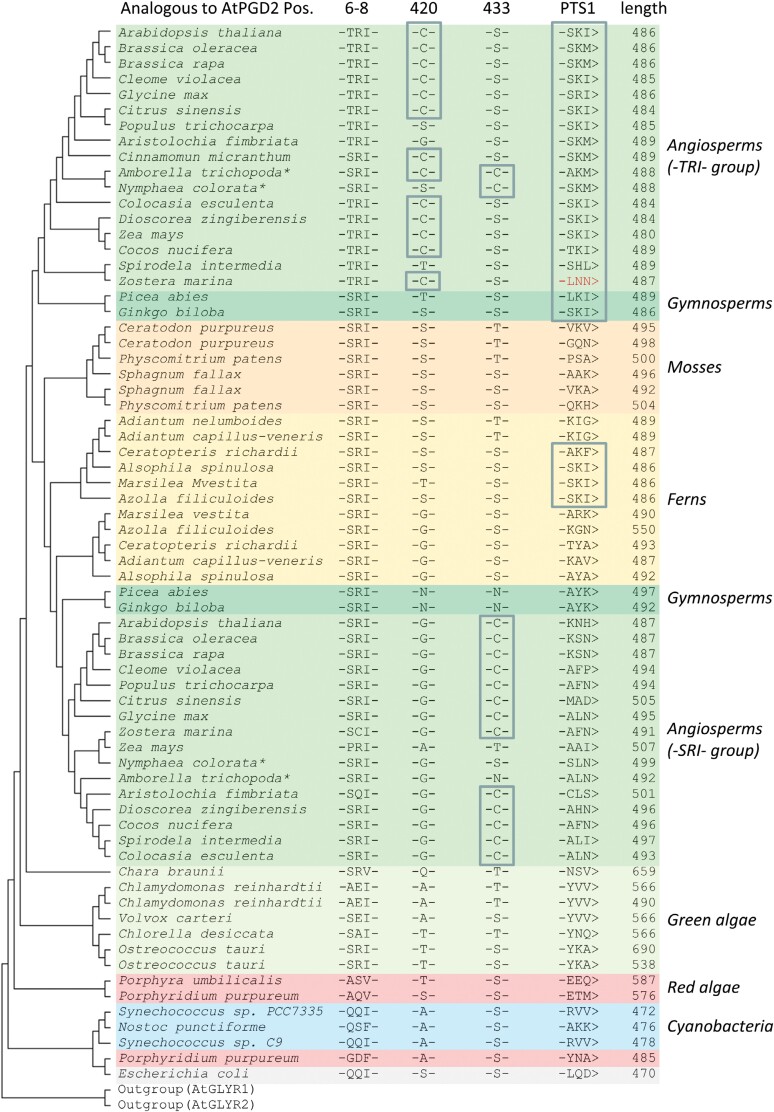
Phylogenetic analyses support a role for PGD2 in peroxisomes of seed plants. The tree was built with amino acid sequences selected from different species. Left, maximum likelihood tree with highest log likelihood (–25 210.27); 70 sequences were compiled with bifunctional glyoxylate reductase isoforms (AtGLYR1/2) as outgroup. Additional features of the PGD sequences are depicted in table format, highlighting relevant positions based on AtPGD2. Note that a PTS1 motif is common among the spermatophyta (first appearing in ferns, yellow background), except for the seagrass *Zostera marina* (red letters) that lives submerged in shallow marine terrain. Asterisks (*) mark the oldest clades of the angiosperms. Subtrees were flipped to present the peroxisomal -TRI- group of *A. thaliana* on top.

PGD2 is needed in peroxisomes during fertilization (Fig. 1), and phosphorylation of a crucial amino acid at the protein’s N-terminus seems to be involved in regulating subcellular localization (Fig. 6) and catalytic activity (Fig. 7). To evaluate these finding for other plant species, we integrated 70 sequences of PGD homologues from members of the land plants and algae by compiling a phylogenetic tree (Fig. 8). As previously shown for angiosperms (Fernández-Fernández and Corpas, 2016), the gymnosperms also group in two clusters, one corresponding to proteins ending with a PTS1 motif that are probably peroxisomal, and the other primarily consisting of plastidial homologues. We extended these analyses by including evolutionarily older species and mapping the additionally identified features. In particular, the position equivalent to phosphomimicry in AtPGD2 and AtPGD1 could be traced back as far as the green algae (-SRI- in *Ostreococcus tauri*), with a few exceptions (-AEI- in *Chlamydomonas reinhardtii*), inferring a conserved function in the green lineage. Furthermore, the emergence of PTS1 motifs was assessed. The first peroxisomal homologues appear in ferns (Fig. 8, highlighted by a frame) and are present in all reviewed gymno- and angiosperms, except for the seagrass *Zostera marina* (-LNN>) that lives and propagates in a submarine environment. The most common PTS1 motifs are canonical (-S/TKI>, -SKI/M>; Chowdhary *et al.*, 2012). In fact, the unique cysteine positions are also conserved among most angiosperms in the two subgroups, with C420 clustering in the peroxisomal (PTS1-containing) -TRI- group, and C433 (equivalent to C435 in AtPGD1) in the plastidial -SRI- group. Most exceptions can be attributed to *Amborella trichopoda* and *Nymphea colorata*, the oldest living angiosperms (Mathews and Donoghue, 1999), indicating a relatively recent evolutionary fixation of peroxisomal localization.

## Discussion

PGD2 has been repeatedly found by peroxisomal proteomic analyses (Reumann *et al.*, 2007, 2009; Eubel *et al.*, 2008; Quan *et al.*, 2013). However, reporter fusions of full-length PGD2 could not be visualized in peroxisomes (Reumann *et al.*, 2007; Hölscher *et al.*, 2016), questioning true dual localization of the enzyme. We first confirmed the importance of peroxisomal 6PGDH activity for fertilization by a genomic approach. In fact, the weak non-canonical PTS1 motif -SEI> (Chowdhary *et al.*, 2012) sufficed to revoke the fertilization block (Fig. 1). This dispelled remaining doubts about PGD2 in peroxisomes, which were raised by localization studies that were only successful with N-terminal truncations that rendered the protein inactive (Hölscher *et al.*, 2016). Yet, these findings pointed to the importance of the N-terminus for a tight regulation of peroxisomal import, somehow preventing recognition of PGD2’s C-terminal canonical PTS1 motif (-SKI>) under most ‘non-stimulated’ conditions.

We proceeded to evaluate the potential importance of Trx_*m2*_ that is involved in peroxisomal import of plastidial OPPP enzymes G6PD1 (Meyer *et al.*, 2011) and PGL3 (Hölscher *et al.*, 2014) by mediating cytosolic retention under oxidative transients. Although we could demonstrate interaction with Trx_*m2*_ for all Arabidopsis PGD isoforms using Y2H and BiFC analyses (Fig. 2), localization changes were not observed—not even for the plastidial isoforms (PGD1/3). Since PGD2 does not target plastids (Lutterbey and von Schaewen, 2016), the significance of its interaction with Trx_*m2*_ remained obscure. Previous reports identified spinach PGD by a Trx_*m*_-trapping approach using isolated chloroplasts (Balmer *et al.*, 2003), and Arabidopsis PGD1 was redox labelled upon H_2_O_2_ treatment (Wang *et al.*, 2012). Interestingly, the C292S and C435S changes in PGD1 resulted in higher rather than lower enzymatic activity (Supplementary Fig. S4). This excludes a role in diurnal OPPP regulation, since only plastidial G6PD enzymes were less active upon C–S change of their conserved regulatory cysteines (Wenderoth *et al*., 1997; Wendt *et al*., 2000; Née *et al.*, 2014). Recently, it was shown that Trx_*m*_ plays a role in Arabidopsis root tissues during stress defence (Née *et al.*, 2023). Thus, protein interaction may not be confined to plastidial G6PD2, G6PD3 (Née *et al.*, 2023), and PGL3 (Hölscher *et al.,* 2014), but probably extends to the PGD isoforms, in particular since most plastidial sequences are merely longer than the peroxisomal PGD isoforms, and interaction of PGD1/3 with Trx_*m2*_ was confined to the N-terminus. The latter may protect them from processing in the stroma, which otherwise would render the enzymes inactive—at this point, interaction with further thioredoxins in the stroma cannot be excluded.

Usually, the presence of a canonical PTS1 motif at the C-terminus (such as -SKI> in PGD2) is necessary and sufficient for peroxisomal import of a given protein (including GFP). To explain PGD2’s failure in peroxisomal localization, we aimed to avoid reporter artefacts by devising new medial GFP fusions. The two constructs that we cloned exhibited opposite behaviour in terms of import and activity (Fig. 4B). In a 3D model of PGD2, reporter insertion in the *old medial* construct lies within the dimer interface—73 amino acids upstream of the -SKI> motif (for details, see Supplementary Fig. S7), leading to peroxisomal localization but loss of activity. These results are similar to the medial construct cloned by Eubel *et al.* (2008) in which C-terminal insertion of enhanced GFP (eGFP; about seven amino acids upstream of the -SKI> motif) exposes the PTS1 artificially. This raises questions about the catalytic activity of the fusion protein, since the reporter is located at a similar position to the T-DNA insertion in *pgd2-1*, resulting in an inactive protein (Fig. 1B; Hölscher *et al.*, 2016).

According to the 3D model, reporter insertion in our *new medial* GFP fusion is unlikely to interfere with PGD dimerization. In fact, we were able to measure activity upon GFP-based pull-down from transfected protoplasts, indirectly validating its ability to form dimers. Yet, peroxisomal localization was not observed, independent of the trophic state of the plants used for protoplast preparation (i.e. with or without sucrose in the growth medium; Hölscher *et al.*, 2014). These observations suggest that monomeric versions of PGD2 are preferred for import.

With availability of a near native medial reporter fusion, we investigated the role of the N-terminus for PGD2 localization. This was important, since N-terminal truncation by 15 amino acids resulted in partial localization in peroxisomes (GFP–PGD2_*mat*; Hölscher *et al.*, 2016). Step-wise amino acid deletion identified a clear turning point for peroxisomal targeting (and inactivation) of PGD2, pointing to the importance of position Thr6 (Fig. 5). When compiling PGD sequences of different species—regardless of their predicted localization—we noticed that most carry either a Ser or a Thr at the corresponding position (Fig. 5C), which both may be phosphorylated. Subsequent analyses of phosphomimetic changes in the PGD2_*new medial* construct demonstrated that only T6E and T6D (Fig. 6), but not T6V (or T6M; Supplementary Fig. S6A), result in peroxisomal localization, with very little cytosolic background. Notably, a synonymous change in plastidial PGD1 (S7D) led to a similarly clear targeting switch to plastids, without any cytosolic fraction. Furthermore, mutation of C420S in the dimer interface occasionally enabled peroxisomal import of full-length PGD2, but to a much lesser extent compared with the Thr6 phosphomimetic changes. Beyond that, C420S was found to only slightly affect catalytic activity upon purification from protoplasts (Fig. 7C), thus this amino acid might be involved in initiating PGD2 dimerization for enzyme stabilization, which may represent an additional layer of targeting control. Under stress conditions that are usually accompanied by oxidative bursts and transiently elevated H_2_O_2_ levels in the cytosol, this may lead to oxidative modification of cysteine residues (e.g. by sulfenylation; reviewed in Corpas *et al.*, 2022). Thus, modification of C420 might enhance monomeric import of PGD2 by delaying (or preventing) dimerization. Yet, since the mutated C420S version retained most of its activity, dimerization still seems possible without formation of a disulfide bridge between the monomers. This is also reflected by the finding that cysteines are not absolutely conserved at this position in peroxisomal isoforms of other plant species (Fig. 8).

The percentage of imaged cells with only partial peroxisomal localization (Fig. 6A, WT), as opposed to those with cytosolic or clear peroxisomal pattern, may suffer from observation bias, since we specifically looked for this pattern. Thus, it remains unclear how frequently the wild-type protein really enters peroxisomes, while the difference from the phosphorylation mimics (T6E, T6D) is stunning, especially since the localization change could be reverted just by deleting the -SKI> motif. Our findings therefore show that peroxisomal uptake of PGD2 entirely depends on its PTS1 motif, unlike in other proteins, such as the PGL isoforms that were also imported via piggybacking (Lansing *et al.*, 2020), or the still enigmatic import of plant catalases (reviewed in Baker *et al.*, 2023).

Subsequently, we also introduced the changes into the N-terminally tagged GFP–PGD2 construct and noticed differences compared with the *new medial* fusions: while C420S was not sufficient to alter the localization pattern, T6E occasionally enabled peroxisomal import, but consistently retained a cytosolic fraction (Fig. 6B) and showed residual catalytic activity (Fig. 7). We therefore suppose that phosphorylated Thr6 recruits a partner protein that holds PGD2 in the monomeric conformation. Interaction with T6E might be only partially possible when the PGD2 N-terminus is shielded by a reporter, resulting in occasional dimerization. One promising candidate would be the 14-3-3 protein GRF10/GF14-epsilon (At1g22300), listed as an interaction partner for the Arabidopsis PGD1 and PGD2 isoforms (Swatek *et al.*, 2011), similarly to a rice isoform with cytosolic annotation (Schoonheim *et al.*, 2007). Our findings furthermore suggest that the partner protein can bind phosphorylated and phosphomimetic amino acids, although there are reports on 14-3-3 proteins contradicting (Duby *et al*., 2009) and supporting this possibility (Chan and Gack, 2016). In any case, an as yet unknown kinase seems to phosphorylate the PGD isoforms before dimer formation is initiated, namely either shortly after or during biosynthesis at ribosomes. Notably, co-translational homodimerization is favoured among proteins with a large interaction interface, including components of protein complexes (Badonyi and Marsh, 2022), which may furthermore promote OPPP metabolon formation in the cytosol. In this context, it is interesting that phosphomimicry of a serine residue (conserved among 14-3-3 family members) promoted dimerization of Arabidopsis GRF2/GF14-omega (At1g67300) in the context -ApSWRIL- (Gökirmak *et al.*, 2015), having potential for co-regulation.

**Fig. 9.**
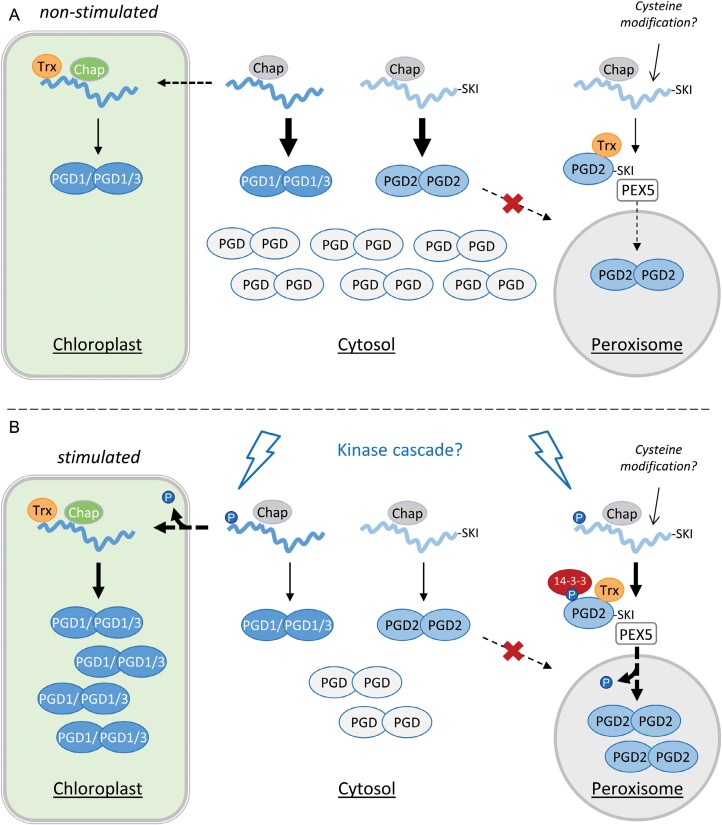
Model of dual targeting regulation by N-terminal PGD phosphorylation. (A) Early homodimerization results in retention of most plastidial PGD1/3 and peroxisomal PGD2 precursors in the cytosol. Binding by chaperons (chap, grey/green) and thioredoxin (Trx, orange) aids in plastid import of PGD1/3 precursors and infrequent peroxisomal uptake of PGD2 monomers (possibly due to cysteine modification/oxidation), as suggested by localization analyses and proteomics data. (B) An unknown stimulus activates kinases (flashes) that phosphorylate the PGD N-termini (blue circles), enforcing uptake into plastids and peroxisomes, respectively (possibly involving a 14-3-3 protein, red). In the target organelles, phosphatase activity is needed to form active PGD dimers. Note that the N-termini of PGD1/3 must not be cleaved upon plastid import (renders them inactive), while the dimerization interface of folded but monomeric PGD2 has to be shielded from unspecific aggregation to enable Pex5 binding to the PTS1 motif (-SKI) for peroxisomal import.

Under normal growth conditions, the crucial N-terminal S/T residues of the PGD isoforms are probably not phosphorylated, leading to a large pool of PGD dimers in the cytosol by default (Fig. 9). We speculate that the C-terminus with the PTS1 motif of dimerized PGD2 is inaccessible for PEX5 (since it is shorter than in sheep 6PGDH resolved by X-ray crystallography at 2.5 Å resolution; Adams *et al.*, 1991), unless chaperons (including thioredoxin disulfide isomerases) are bound to shield the dimer interface, leading to infrequent peroxisomal import. Notably, in our BiFC analyses (with reconstituted YFP halves that are unable to dissociate), Trx_*m2*_ was co-imported into peroxisomes together with PGD2_Δ15 (Fig. 2C, bottom), indicating accessibility of the PTS1 in this constellation. Under certain conditions (stress/oxidative transients in the cytosol), a kinase is activated that leads to early (or even co-translational) phosphorylation of the PGD N-termini, which allows partner proteins (such as 14-3-3) to bind and enables efficient import of monomeric PGD2 subunits via Pex5 into peroxisomes (and of PGD1/3 into plastids). Phosphatase activity at the target locations is needed to enable dimer formation as a prerequisite for catalytic activity of the dehydrogenases, which is also present in peroxisomes (Kataya *et al.*, 2016; Liu *et al.*, 2023). Concerning the need for early/co-translational phosphorylation of the PGD N-termini, known interaction of translating polysomes with the actin cytoskeleton (Davies *et al.*, 1991; Klyachko *et al.*, 2000) that is also involved in mediating the close proximity of plastids with peroxisomes—especially during photosynthesis (Oikawa *et al.,* 2015)—should surely be beneficial for stimulus-induced efficient organelle import (Fig. 9).

In summary, we identified three key components for peroxisomal import of Arabidopsis PGD2 that are highly conserved in seed plants, especially among the angiosperms. We found that the sequences cluster in two distinct groups, with the presence of T6, C420, and a PTS1 motif in the peroxisomal group probably reflecting involvement in a fundamental mechanism. In Hölscher *et al.* (2016), we discussed the importance of peroxisomal PGD2 activity during pollen tube guidance to explain the fertilization defect. In fact, among the plant species examined, we identified only the submarine seagrass *Z. marina* to carry no PTS1 motif, despite featuring conservation of T6 and C420. Interestingly, pollen tube growth of *Z. marina* was described as partly disorganized (Ackerman, 1993), which matches the previously observed erratic pollen tube growth in ovaries of heterozygous *PGD2 pgd2-1* mutant plants (Hölscher *et al.*, 2016). Moreover, this hints at a conserved role for the PGD2 homologues (and the entire OPPP) in peroxisomes during funicular guidance, not only in Arabidopsis, but probably in all flowering plants. Thus, we will try to overcome the fertilization block in Arabidopsis, which might yield viable *pgd2-1* mutant plants and allow us to study the role of PGD2 also during vegetative growth.

All in all, our data show that protein import into peroxisomes can be more complex than the presence of a canonical PTS1 motif may suggest, when subject to post-translational modification(s). Physiologically, C420 modification would facilitate peroxisomal import of PGD2 under mildly or non-stressed conditions, explaining the basal levels found by peroxisomal proteomics (Reumann *et al.*, 2007; Eubel *et al.*, 2008; Quan *et al.*, 2013), while N-terminal phosphorylation (upon a specific stimulus) would increase organelle import drastically. Our results suggest exclusive import of folded PGD2 monomers, which is in line with earlier reports on catalase subunits and other peroxisomal proteins in mammalian cells (Freitas *et al.*, 2015). In fact, tetramerization of catalase was recently shown to be regulated by de-phosphorylation in rice peroxisomes (Liu *et al.*, 2023). This is strong additional support for our findings, with the cytosolic kinase responsible for the complete localization shift remaining to be identified. So far, a search with the PGD2 target sequence -X-P-pT-R-X- in PhosPhAt 4.0 found several proteins with this signature. Whether some of them may be linked to targeting of OPPP-relevant enzymes to peroxisomes (e.g. during fertilization) remains to be investigated. Other sequentially acting Ser/Thr kinases may be involved as well, such as MPK3 and MPK6 that were shown to be important during funicular pollen tube guidance (Guan *et al.*, 2014) and for *GPT1* expression via WRKY transcription factors (Zheng *et al.*, 2018), notably with a link to sugar signaling via d-glucose (Bhagat *et al.*, 2022). To elucidate possible further connections with our findings will be an important task for the near future.

## Supplementary data

The following supplementary data are available at *JXB* online.

Table S1. Oligonucleotides used in this study.

Fig. S1. *In vitro* activity of the His-PGD2 variants upon purification from *E. coli*.

Fig. S2. Single channel images of Fig. 2B and C.

Fig. S3. Single channel images of Fig. 3A and B.

Fig. S4. Analysis of the conserved cysteines unique to PGD1 and PGD3.

Fig. S5. Single channel images of Fig. 5B and further supporting co-expression analyses.

Fig. S6. Single channel images of Fig. 6A.

Fig. S7. Amino acid alignment of the Arabidopsis PGD isoforms.

erae077_suppl_Supplementary_Figures_S1-S7_Table_S1

## Data Availability

Biological materials and data not shown in the supplementary data can be made available on request.

## Abbreviations

BiFC: bimolecular fluorescent complementation
ER: endoplasmic reticulum
G6P: glucose-6-phosphate
G6PD(H): glucose-6-phosphate dehydrogenase
GPT: glucose-6-phosphate transporter
GFP: green fluorescent protein
OFP: orange-shifted monomeric red fluorescent protein
OPPP: oxidative pentose-phosphate pathway
6PGDH/PGD: 6-phosphogluconate dehydrogenase
PGL: 6-phosphogluconolactonase
PTS: peroxisomal targeting signal
Ru5P: ribulose-5-phosphate
Trx: thioredoxin
YFP: yellow fluorescent protein
