# Supplementary material for: Evidence for dual targeting control of Arabidopsis 6-phosphogluconate dehydrogenase isoforms by N-terminal phosphorylation
Source: J Exp Bot. 2024 Feb 27;75(10):2848–66. doi: 10.1093/jxb/erae077 (PMC11103113; doi:10.1093/jxb/erae077)
Supplement: erae077_suppl_Supplementary_Figures_S1-S7_Table_S1 [file erae077_suppl_supplementary_figures_s1-s7_table_s1.pdf]

Table S1. Oligonucleotides used in this study.

Lab no.	Name	Sequence	Construct and Application
PGD1			
753	PGD1 SpeI s	NNACTAGTATGGAGTCCGCCGCACTATC	YFP$^{N/C}$-PGD1/PGD1- YFP$^{N/C}$
592	PGD1 +Stop BamHI as	NNNGGATCCTCAATGGTTCTTCCTGGCAA	YFP$^{N/C}$-PGD1/pET-PGD1_Δ6
773	PGD1 -Stop BamHI as	NNNGGATCCATGGTTCTTCCTGGCAA	PGD1- YFP$^{N/C}$
499	PGD1 -Stop NcoI as	NNNCCATGGNATGGTTCTTCCTGGCAAGCTTAGTC	PGD1-GFP
1784	PGD1 SDM S7D s	ATGGAGTCCGCCGCTCTAGACCGAATCGG	PGD1 S7D-GFP
1785	PGD1 SDM S7D as	GACCGATTCGGTCTAGAGCGGCGGACTC	PGD1 S7D-GFP
1786	PGD1 SDM S7A s	GGAGTCCGCAGCGCTAGCCCGAATCGG	PGD1 S7A-GFP
1787	PGD1 SDM S7A as	CCGATTCGGGCTAGCGCTGCGGACTCC	PGD1-GFP
1376	PGD1 Δ6 NdeI s	NNNCATATGCGAATCGGTCTCGCC	pET-PGD1_Δ6
1482	PGD1 SDM C292 s	GCTGCGTCGTTGGATTCTAGATACTTGAGTG	pET-PGD1_Δ6 C292S
1483	PGD1 SDM C292 as	CACTCAAGTATCTAGAATCCAACGACGCAGC	pET-PGD1_Δ6 C292S
1484	PGD1 SDM C435S s	ACGCCTGGAATGAGCGCTAGTCTTGCGTAT	pET-PGD1_Δ6 C435S
1485	PGD1 SDM C435 as	ATACGCAAGACTAGCGCTCATTCCAGGCGT	pET-PGD1_Δ6 C435S
PGD2			
674	*gPGD2* SDM ΔSKI s	GATTGCAAGACAATGAAAGATCTGAGATCTC	Compl. construct *gPGD2*_ΔSKI/pET-PGD2_ΔSKI
675	*gPGD2* SDM ΔSKI as	GAGATCTCAGATCTTTCATTGTCTTGCAATC	Compl. construct *gPGD2*_ΔSKI/pET-PGD2_ΔSKI
448	*gPGD2* SDM SEI s	GATTGCAAGACAATCTGAGATCTGAGATCTCTCATCC	Compl. construct *gPGD2*_SEI/pET-PGD2_SEI
449	*gPGD2* SDM SEI as	GGATGAGAGATCTCAGATCTCAGATTGTCTTGCAATC	Compl. construct *gPGD2*_SEI/pET-PGD2_SEI
486	PGD2 full NdeI s	NNNCATATGGCTGTTCAACCTACAAGAATAG	Y2H(BD)/all pET-PGD2 variants
487	PGD2 BamHI as	NNNGGATCCTCAGATCTTAGATTGTCTTGCAATC	PGD2_*new medial* (C-term)/ pET-PGD2 (+variants)/ Y2H(BD)
695	PGD2_Δ15 NdeI s	NNNCATATGGGTCAGAACCTAGCTCTCAAC	Y2H(BD)
535	PGD2 SpeI s	NNNACTAGTATGGCTGTTCAACCTACAAG	YFP$^{N/C}$-PGD2/PGD2- YFP$^{N/C}$
536	PGD2 +Stop SalI as	NNNGTCGACGATCTTAGATTGTCTTG	YFP$^{N/C}$-PGD2
537	PGD2 -Stop SalI as	NNNGTCGACGATCTTAGATTGTCTTG	PGD2- YFP$^{N/C}$
706	PGD2_Δ15 SpeI s	NNNACTAGTATGGGTCAGAACCTAGCTCTC	PGD2_*new medial*_Δ15/ YFP$^{N/C}$-PGD2_Δ15/ PGD2_Δ15- YFP$^{N/C}$
1306	PGD2 SDM C420S s	GAGAAGAGTCGTCTCCTTAGCTATCAACTC	YFP$^{N/C}$-PGD2_Δ15 C420S/ PGD2_*new medial* C420S/ mGFP-PGD2 C420S
1307	PGD2 SDM C420S as	GAGTTGATAGCTAAGGAGACGACTCTTCTC	YFP$^{N/C}$-PGD2_Δ15 C420S/ PGD2_*new medial* C420S/ mGFP-PGD2 C420S
62	PGD2 XbaI s	NNNTCTAGACATGGCTGTTCAACCTACAAGAATAG	PGD2_*new medial* (N-term)/ PGD2_*old medial* (N-term)
1143	PGD2 N-newmed NcoI as	NNNCCATGGAGGTCAAGATATCTCCAAACCC	PGD2_*new medial* (N-term) (+Δx variants)
1142	PGD2 C-newmed SpeI s	NNNACTAGTGCAGATCAGAAAGTTGACAAGAAACAGC	PGD2_*new medial* (C-term)
63	PGD2 N-med XhoI as	NNNCTCGAGTCTCTCGATTATCTCTTTCGCAAAC	PGD2_*old medial* (N-term)
103	PGD2 C-med SpeI s	GAGATAATCACTAGTACAGTCCGCGTGGAGAAG	PGD2_*old medial* (C-term)
107	PGD2 XhoI as	NNNNCTCGAGTCAGATCTTAGATTGTCTTGC	PGD2_*old medial* (C-term)
1685	PGD2_Δ4 SpeI s	NNNACTAGTATGCCTACAAGAATAGGTCTAGC	PGD2_*new medial*Δ4
1341	PGD2_Δ5 SpeI s	NNNACTAGTATGAGAATAGGTCTAGCTGGACTTG	PGD2_*new medial*Δ5
1769	PGD2 SDM T6V s	GGCTGTTCAACCTGTAAGAATAGGTCTAGC	PGD2_*new medial* T6V
1770	PGD2 SDM T6V as	GCTAGACCTATTCTTACAGGTTGAACAGCC	PGD2_*new medial* T6V

Table S2 continued. Oligonucleotides used in this study.

Lab no.	Name	Sequence	Construct and Application
1771	PGD2 SDM T6D s	GGCTGTTCAACCTGACAGAATAGGTCTAGC	PGD2_*new medial* T6D
1772	PGD2 SDM T6D as	GCTAGACCTATTCTGTCAGGTTGAACAGCC	PGD2_*new medial* T6D
1773	PGD2 SDM T6E s	GGCTGTTCAACCTGAAAGAATAGGTCTAGC	PGD2_*new medial* T6E/mGFP-PGD2 T6E/pET-PGD2 T6E
1774	PGD2 SDM T6E as	GCTAGACCTATTCTTTCAGGTTGAACAGCC	PGD2_*new medial* T6E/ mGFP-PGD2 T6E/pET-PGD2 T6E
1777	PGD2 SDM T6M s	GGCTGTTCAACCTATGAGAATAGGTCTAGC	PGD2_*new medial* T6M
1778	PGD2 SDM T6M as	GCTAGACCTATTCTCATAGGTTGAACAGCC	PGD2_*new medial* T6M
***PGD2* genotyping**			
173	SALK 071687 RP	AGCAAGCTGCTGAGCTATCTG	PGD2 WT
185	SALK 071687 LP	GCCGTACGAGAATTCGGTAAC	PGD2 WT
445	PGD2 WT as	CATAGCTGCTGAGATCAGTTTC	PGD2 WT (does not bind comp. constructs)
	LBa1	TGGTTCACGTAGTGGGCCATCG	Salk TDNA
PGD3			
755	PGD3 SpeI s	NNACTAGTATGGAGTCCGTCGCTCTATC	$YFP^{N/C}$-PGD3/PGD3- $YFP^{N/C}$
594	PGD3 +Stop BamHI as	NNNGGATCCTTACTGACTCTTCCTTGCAAG	$YFP^{N/C}$-PGD3
773	PGD3 -Stop BamHI as	NNNGGATCCCTGACTCTTCCTTGCAAG	PGD3- $YFP^{N/C}$
Trx_{m2}			
517	Trx m2 sense SpeI	NNNACTAGTATGGCTGCTTTCACTTG	$YFP^{N/C}$-Trx m2/ Trx m2- $YFP^{N/C}$
518	Trx m2 as BamHI	GGATCCTCATGGCAAGAACTTGTC	$YFP^{N/C}$-Trx m2
434	Trx m2 as -Stopp XhoI	NNNNNNCTCGAGTGGCAAGAACTTGTC	Trx m2- $YFP^{N/C}$

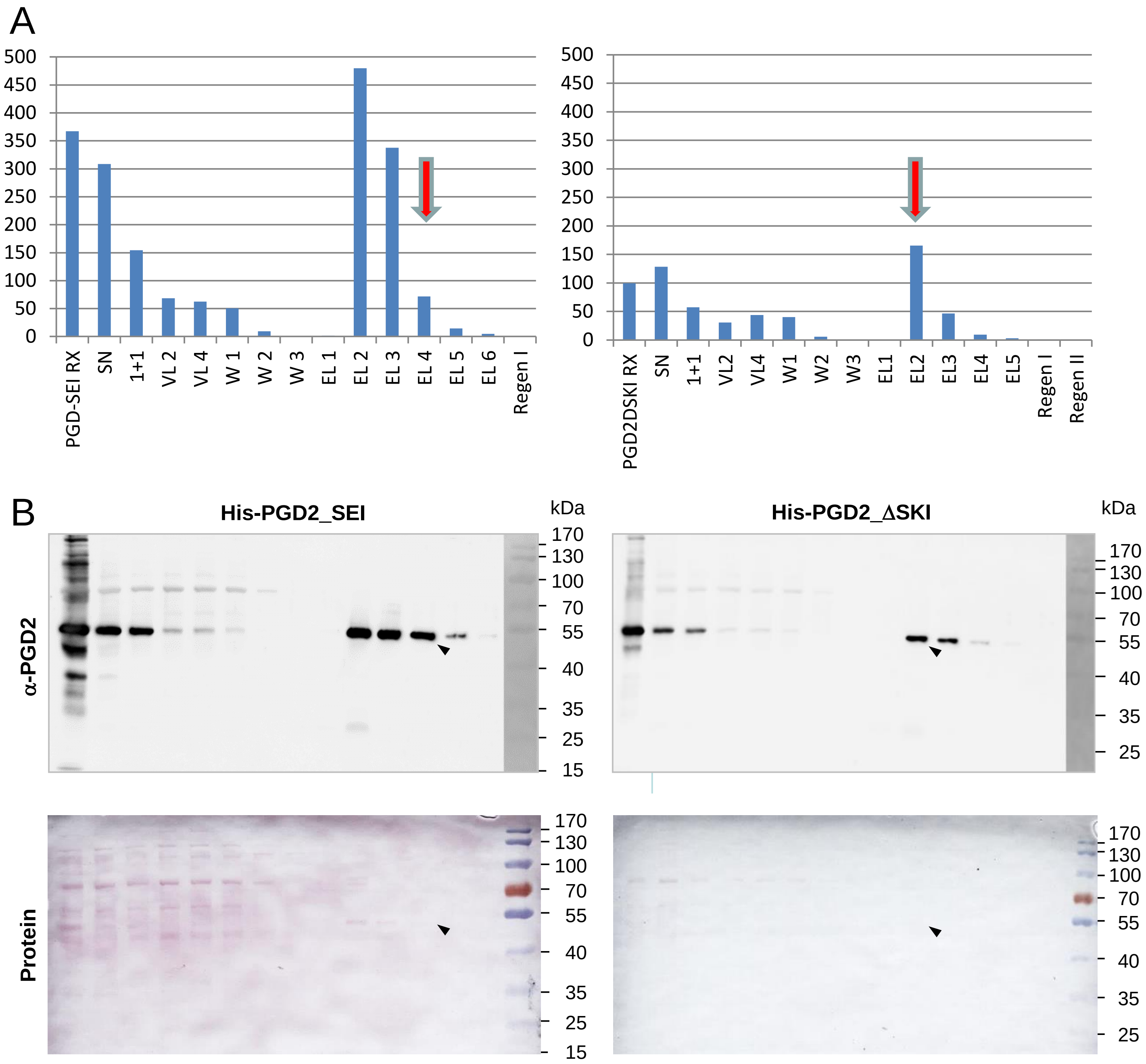

Figure S1. In vitro activity of the His-PGD2 variants upon purification from *E. coli*.

A, Volume activities (nmol min^{-1} ml^{-1}) of different fractions during purification of His-PGD2-SEI and His-PGD2_ΔSKI from *E. coli* strain BL21^{minus} (Meyer et al., 2011) via Ni-NTA columns (Qiagen). RX, crude extract (after sonification); SN, supernatant (after centrifugation); 1+1, dilution with binding buffer; VL, flow-through; W, wash fractions; EL, elution fractions; Regen, after column regeneration (steps I and II).

B, Aliquots (8 µl) were used for SDS-PAGE separation and blot transfer. The Ponceau S-stained blot (protein) was developed with PGD2 antiserum (α-PGD, 1:15,000; as in Hölscher et al., 2016). Molecular masses in kDa, PageRuler™ Prestained Protein Ladder (Fermentas). Fractions with similar signal strength (arrows) were used for the immunoblots shown in Figure 1B of the main manuscript. For purification of His-PGD2 and His-PGD2-1 see Suppl. Figure S3 of Hölscher et al., 2016.

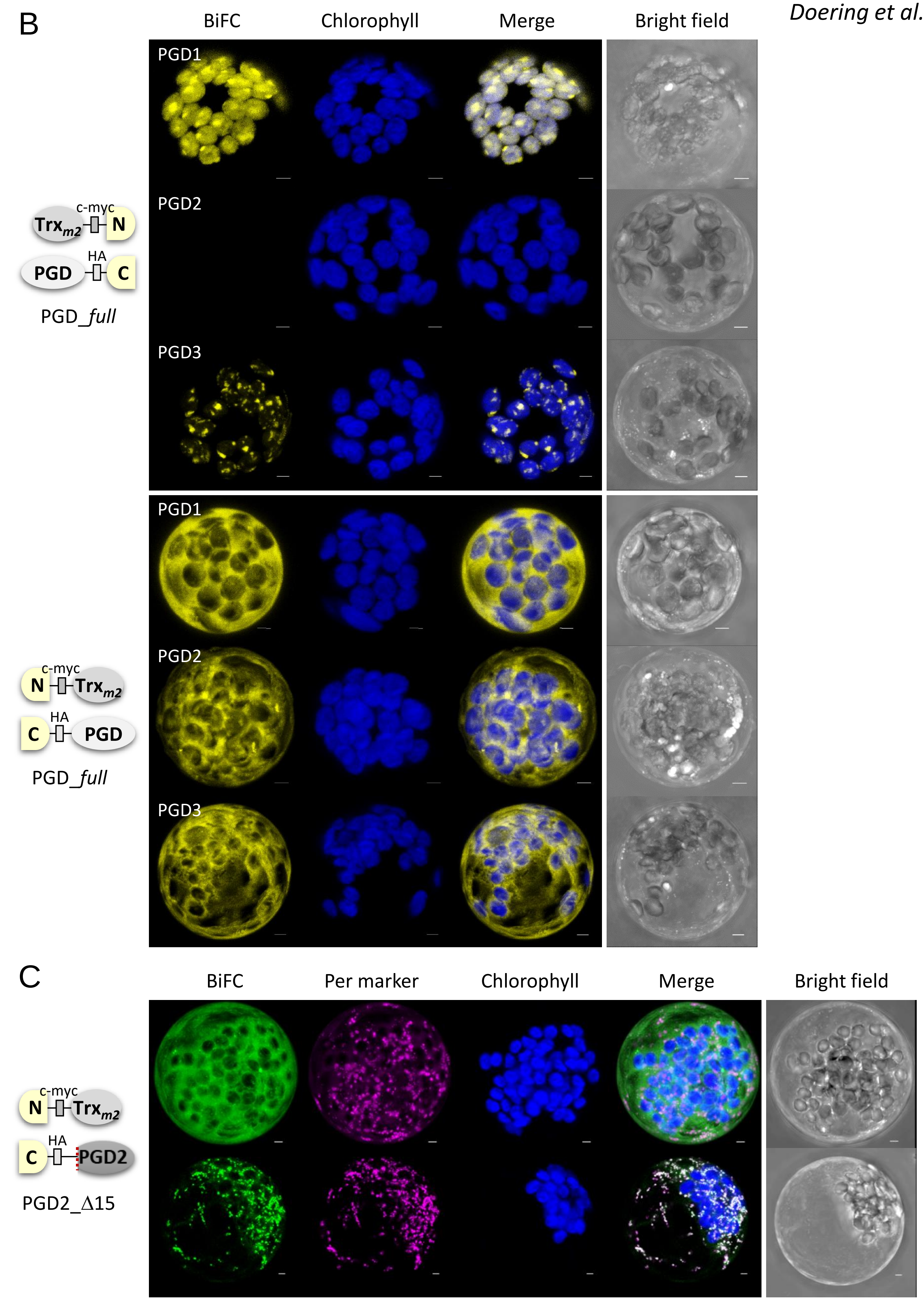

Figure S2. Single channel images of Figure 2B and 2C.
Bright field images are shown as reference. Scale bars 3 µm.

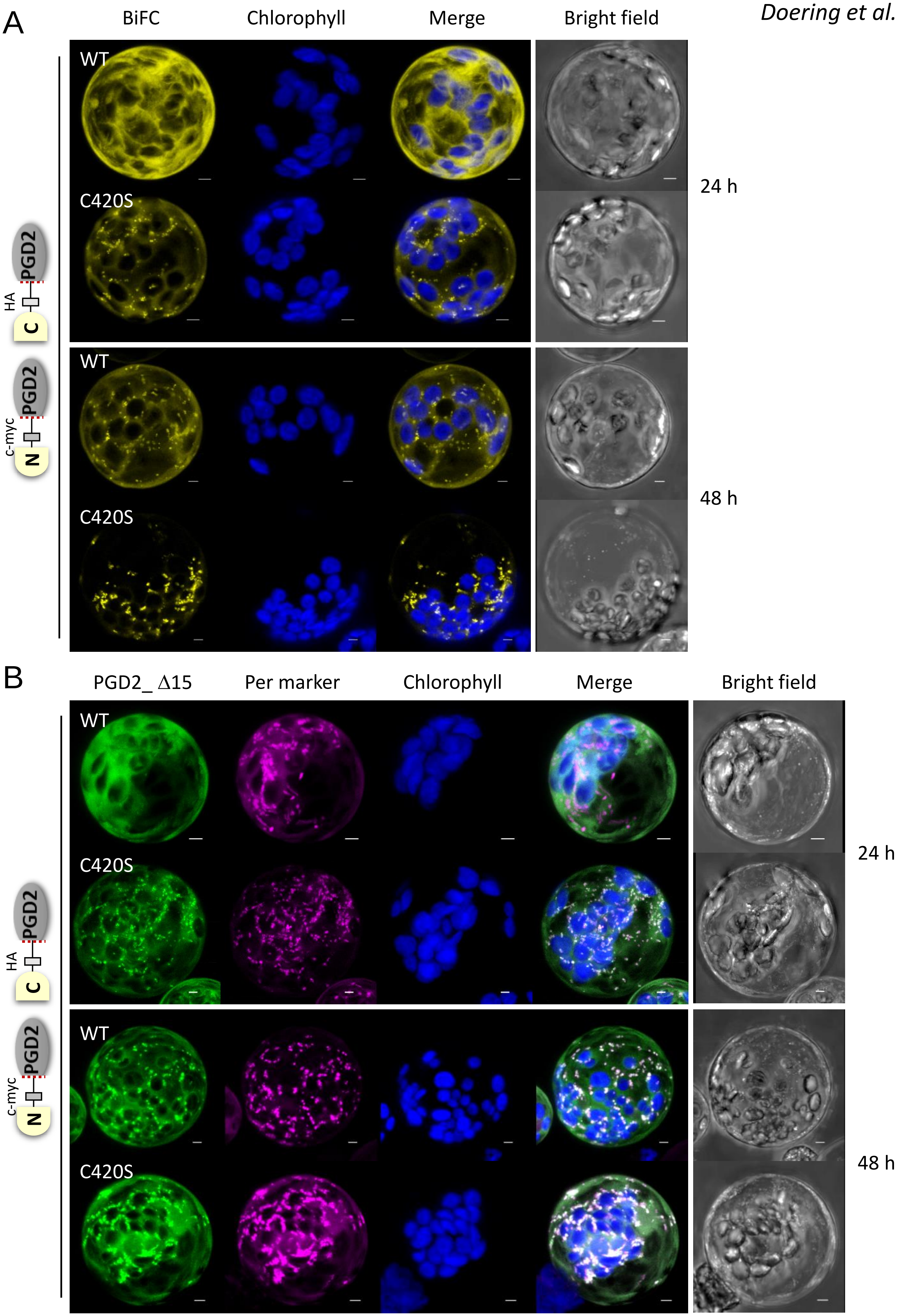

Figure S3. Single channel images of Figure 3A and 3B.
Bright field images are shown as reference. Scale bars 3 µm.

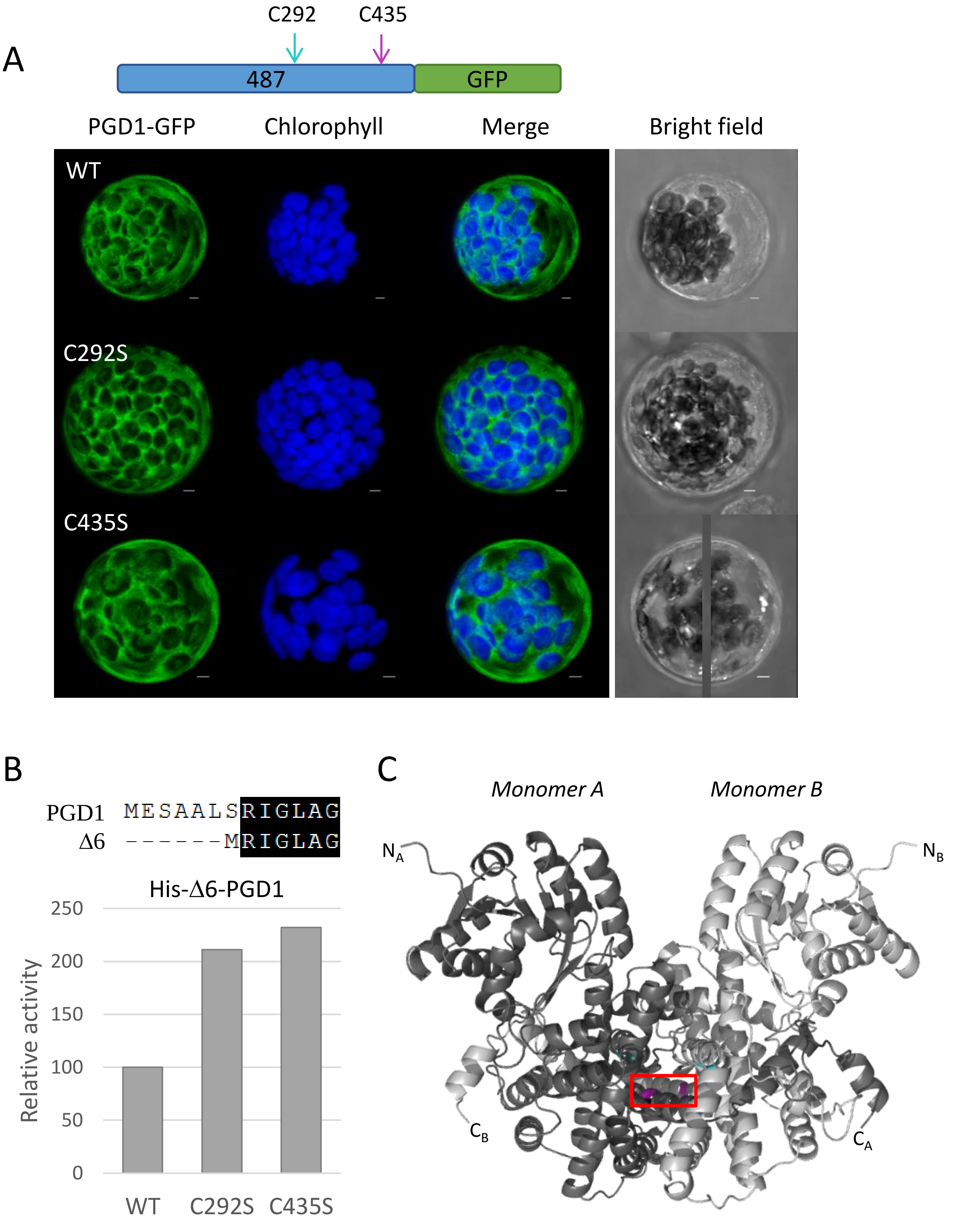

Figure S4. Analysis of the conserved cysteines unique to PGD1 and PGD3.

A, Two cysteines are unique to PGD1/3, but exchange for serine did not alter the localization of PGD1-GFP in Arabidopsis protoplasts (48 h post transfection). All images show maximal projections of ca. 35 single sections. GFP fusions in green, chlorophyll autofluorescence in blue. Colocalization of green and chlorophyll in the merge, or very close signals (<200 nm), appear whitish. Bright field images are shown as reference. Scale bars 3 µm.

B, Relative activity of the C292S and C435S versions compared to His-Δ6-PGD1 wildtype (WT) immunoblot signals upon purification from *E. coli* BL21^{minus} (not shown).

C, PGD1 monomer and dimer showing that both cysteines unique to PGD1/3 lie at the dimer interface, but only C435 on opposing sides (for extent of conservation in the Angiosperms, see PGD2 Pos. 433 in Figure 8).

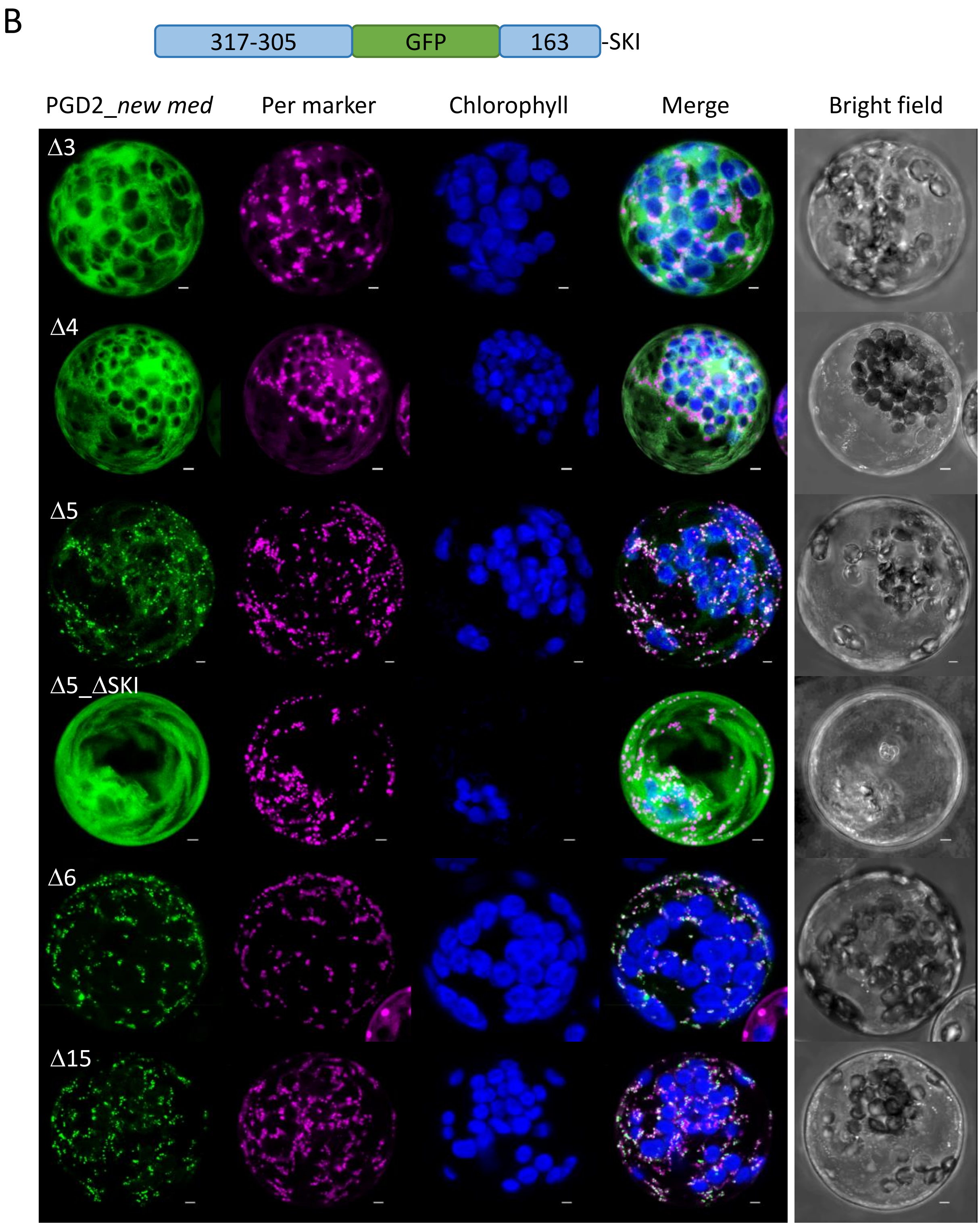

Figure S5. Single channel images of Figure 5B and further supporting co-expression analyses. Localization of N-terminally truncated PGD2_*new medial* versions in Arabidopsis protoplasts isolated from leaves (48 h post transfection). PGD2 fusions in green, peroxisomal marker (OFP-PGL3_*C-short*) in magenta, chlorophyll autofluorescence in blue. Colocalization of green and magenta in the merge, or very close signals (<200 nm), appear white. Bright field images are shown as reference. Scale bars, 3 µm.

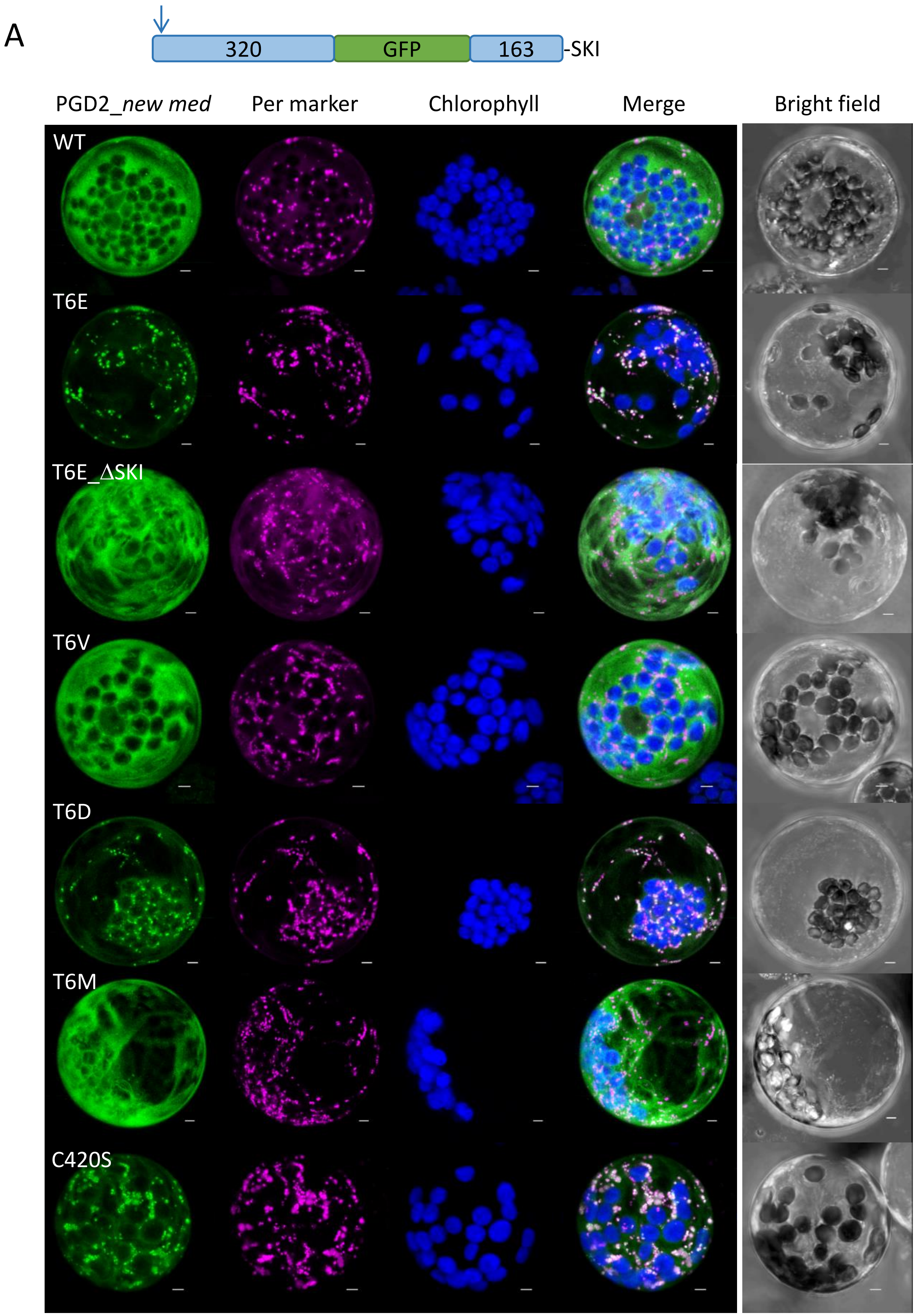

Figure S6. Single channel images of Figure 6A.
Bright field images are shown as reference. Scale bars 3 µm.

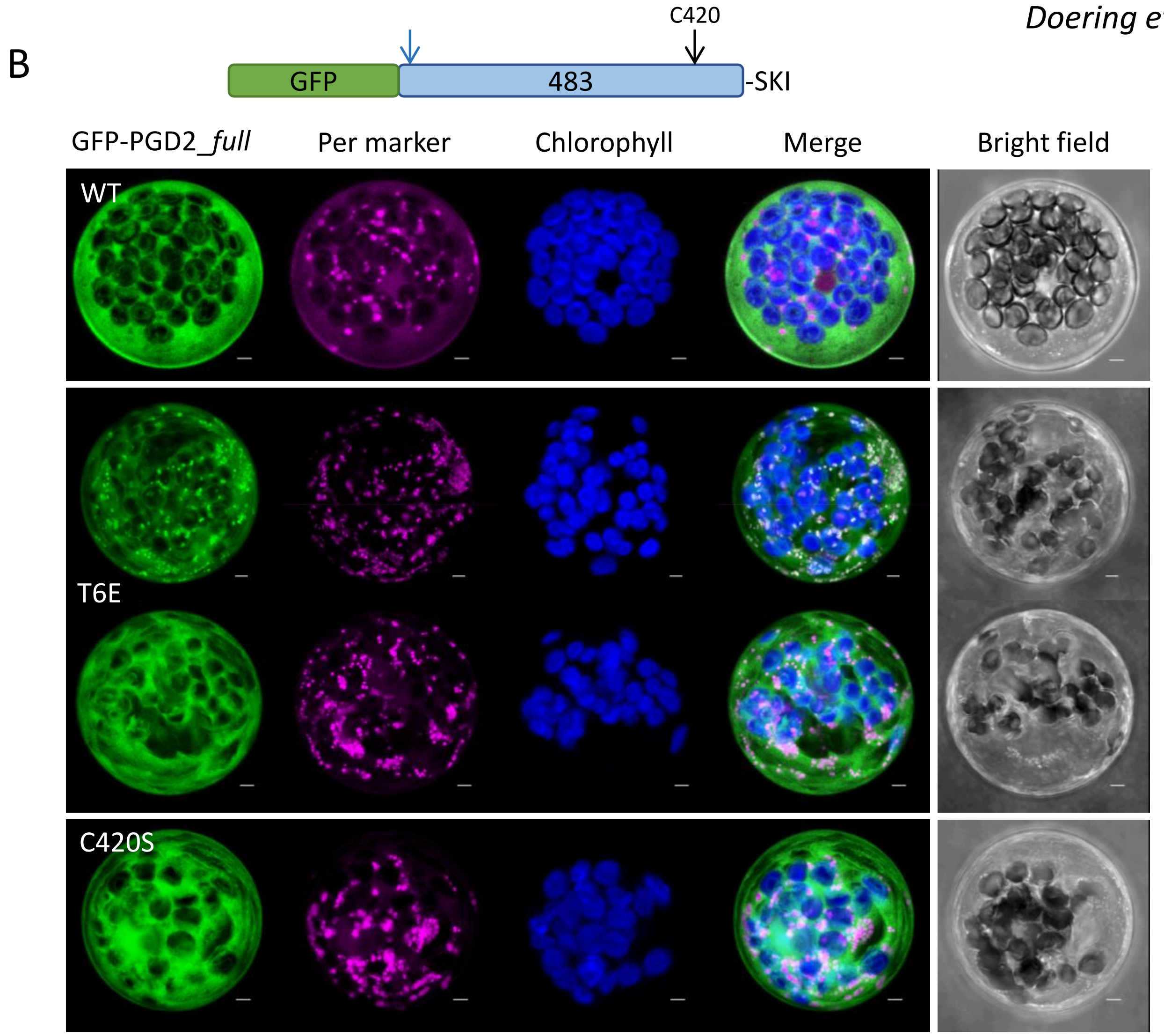

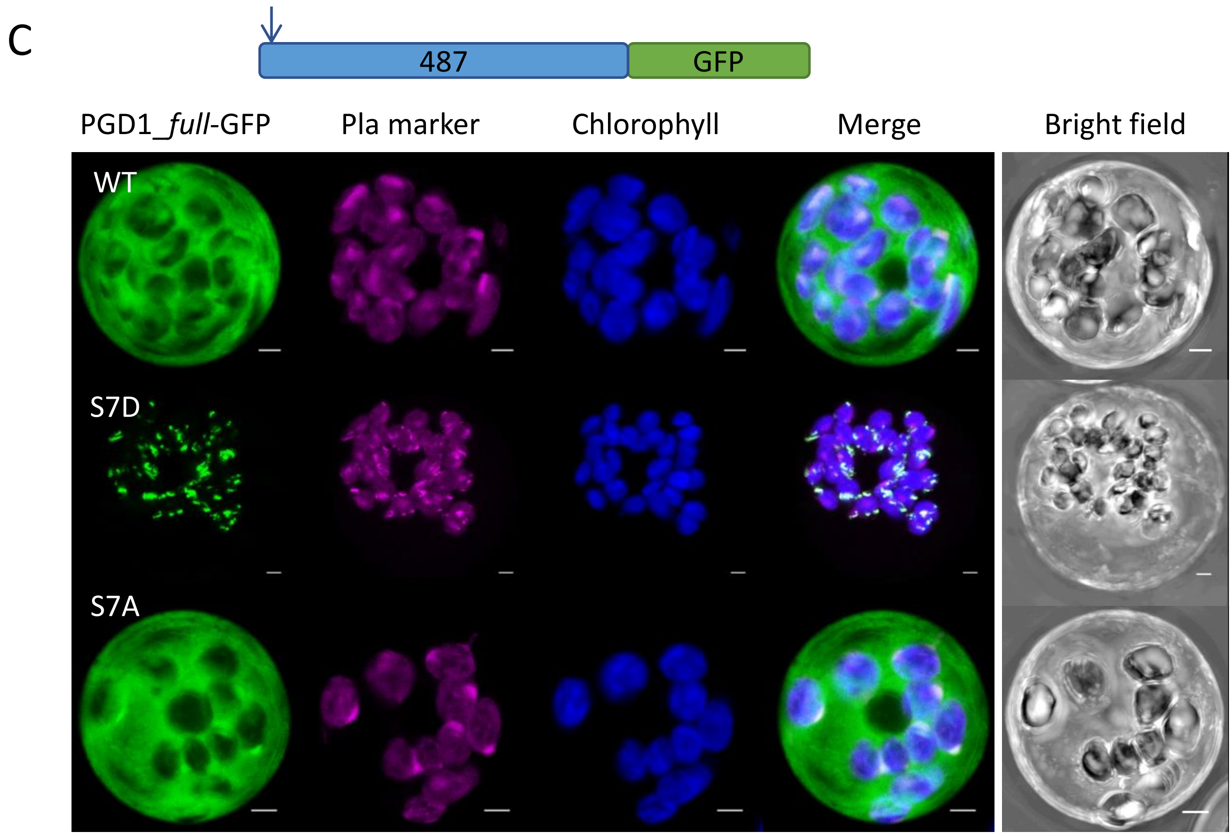

Figure S6. (continued) Single channel images of Figure 6B and 6C.
Bright field images are shown as reference. Scale bars 3 µm.

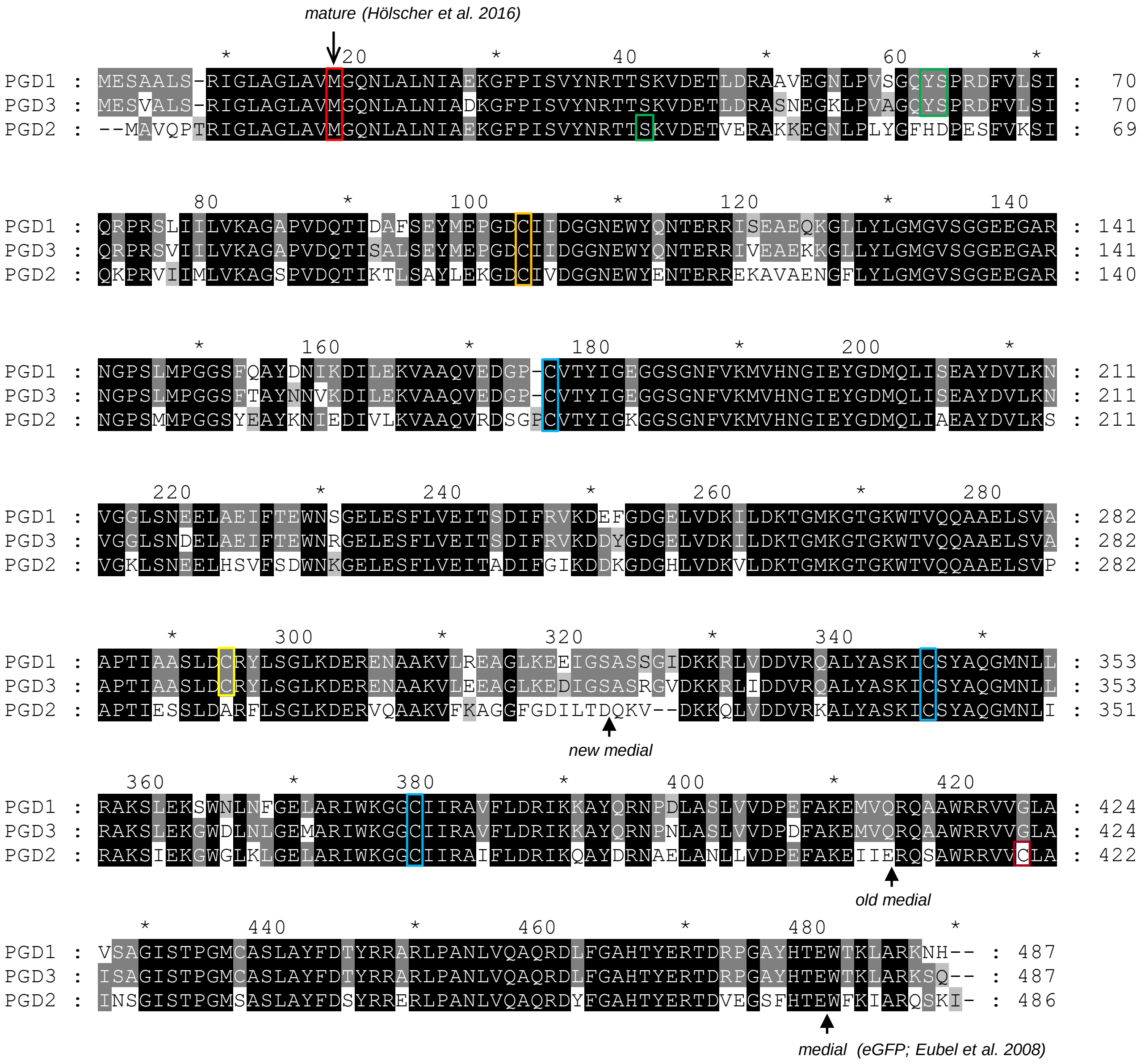

Figure S7. Amino-acid alignment of the Arabidopsis PGD isoforms.
PGD1 and PGD3 are plastidial isoforms, but similar length with PGD2 allowed no prediction on localization. With C-terminal GFP, they showed dual localization (cytosolic/plastididal), also when including the 5'UTR or mutation of the 2nd methionine (red frame; Hölscher et al., 2016). Insertion positions of the PGD2 *medial* GFP fusions are indicated by closed arrows. Concerning the cysteine positions, three are shared with the cyanobacterial isoforms (cyan frames), and one is common to the Arabidopsis PGD isoforms (orange frame). Cysteines C292 and C435 are unique to PGD1 and PGD3 (yellow frame), whereas C420 is unique to PGD2 (dark red). Note that PhosPhAt 4.0 lists S39 for PGD2 and Y61/S62 as phosphorylation sites (green frames).

References

Eubel H, Meyer EH, Taylor NL, Bussell JD, O'Toole N, Heazlewood JL, Castleden I, Small ID, Smith SM, Millar AH. 2008. Novel proteins, putative membrane transporters, and an integrated metabolic network are revealed by quantitative proteomic analysis of Arabidopsis cell culture peroxisomes. Plant Physiology. 148(4):1809–1829. doi:10.1104/pp.108.129999.

Hölscher C, Lutterbey M-C, Lansing H, Meyer T, Fischer K, von Schaewen A. 2016. Defects in Peroxisomal 6-Phosphogluconate Dehydrogenase Isoform PGD2 Prevent Gametophytic Interaction in *Arabidopsis thaliana*. Plant Physiology. 171(1):192–205. doi:10.1104/pp.15.01301.

Meyer T, Hölscher C, Schwöppe C, von Schaewen A. 2011. Alternative targeting of Arabidopsis plastidic glucose-6-phosphate dehydrogenase G6PD1 involves cysteine-dependent interaction with G6PD4 in the cytosol. The Plant Journal. 66(5):745–758. doi:10.1111/j.1365-313X.2011.04535.x.
